# Investigation on Data Fusion of Multisource Spectral Data for Rice Leaf Diseases Identification Using Machine Learning Methods

**DOI:** 10.3389/fpls.2020.577063

**Published:** 2020-11-10

**Authors:** Lei Feng, Baohua Wu, Susu Zhu, Junmin Wang, Zhenzhu Su, Fei Liu, Yong He, Chu Zhang

**Affiliations:** ^1^College of Biosystems Engineering and Food Science, Zhejiang University, Hangzhou, China; ^2^Key Laboratory of Spectroscopy Sensing, Ministry of Agriculture and Rural Affairs, Hangzhou, China; ^3^Institute of Crop Science and Nuclear Technology Utilization, Zhejiang Academy of Agricultural Sciences, Hangzhou, China; ^4^State Key Laboratory for Rice Biology, Institute of Biotechnology, Zhejiang University, Hangzhou, China

**Keywords:** hyperspectral imaging, mid-infrared spectroscopy, laser-induced breakdown spectroscopy, data fusion, rice disease

## Abstract

Rice diseases are major threats to rice yield and quality. Rapid and accurate detection of rice diseases is of great importance for precise disease prevention and treatment. Various spectroscopic techniques have been used to detect plant diseases. To rapidly and accurately detect three different rice diseases [leaf blight (*Xanthomonas oryzae* pv. *Oryzae*), rice blast (*Pyricularia oryzae*), and rice sheath blight (*Rhizoctonia solani*)], three spectroscopic techniques were applied, including visible/near-infrared hyperspectral imaging (HSI) spectra, mid-infrared spectroscopy (MIR), and laser-induced breakdown spectroscopy (LIBS). Three different levels of data fusion (raw data fusion, feature fusion, and decision fusion) fusing three different types of spectral features were adopted to categorize the diseases of rice. Principal component analysis (PCA) and autoencoder (AE) were used to extract features. Identification models based on each technique and different fusion levels were built using support vector machine (SVM), logistic regression (LR), and convolution neural network (CNN) models. Models based on HSI performed better than those based on MIR and LIBS, with the accuracy over 93% for the test set based on PCA features of HSI spectra. The performance of rice disease identification varied with different levels of fusion. The results showed that feature fusion and decision fusion could enhance identification performance. The overall results illustrated that the three techniques could be used to identify rice diseases, and data fusion strategies have great potential to be used for rice disease detection.

## Introduction

With the increase of population, the demand for food supply will surge. To meet such a great need of food, it is critical to improve crop efficiency to increase the food supply. Cereals are stable food supply for human beings. Due to the changes in climate and environment, biological and abiotic stresses which hinder the normal growth of crops become increasingly frequent. The disease is one of the major stresses of crops, causing severe losses in quality and yield (Skolik et al., 2019; Yang et al., 2019).

Rice is one of the most popular staple food sources in the world, and rice is widely planted all over the world, especially in Asia and Africa. However, there are various diseases influencing rice growth. Bacterial leaf blight (*Xanthomonas oryzae* pv. *Oryzae*) (Alberto, 2018), blast (*Pyricularia oryzae*) (Gaoqiang et al., 2020), and sheath blight (*Rhizoctonia solani*) (Yuan et al., 2019) are the three major diseases of rice (Kumar et al., 2020; Molla et al., 2020). Prevention and treatment of disease is an indispensable task for rice growth management at the current time. Traditionally, on the one hand, detection of rice diseases is mainly based on the experts or experienced farmers, with their visual and manual work. On the other hand, with the development of molecular biology and the related techniques, rice diseases can be accurately detected, and these techniques have been widely used as “standard” or “reference” techniques in the related fields. The shortcomings of these techniques are also obvious. They are time consuming, expensive, and complex to be operated.

Rapid and accurate techniques for rice disease detection are of great importance for rice growth management. For the past decades, optical characteristics of plants have widely been studied (Carter and Knapp, 2001; Altangerel et al., 2017; Ribeiro et al., 2018; Liu et al., 2019; Zahid et al., 2019). Under the stress of diseases, external features such as morphology, color, and texture are changed. The plants’ self-defense systems also work to alleviate the damage, resulting in the changes of physiological and biochemical parameters. These changes can be captured by various spectroscopic techniques based on different principals. Researchers have used various spectroscopic techniques for plant disease detection (Zhang et al., 2017; Thomas et al., 2018; Farber et al., 2019; Liu et al., 2019). In this study, to detect rice diseases, spectral information of visible/near-infrared hyperspectral imaging (HSI), mid-infrared spectroscopy (MIR), and laser-induced breakdown spectroscopy (LIBS) were used.

HSI integrates both visible/near-infrared spectroscopy and imaging techniques. Visible/near-infrared spectroscopy has a strong relationship with biological and physiological parameters and internal structures of plants, and it is the most widely used spectroscopic technique to monitor plant growth and plant stresses (Knauer et al., 2017; Asaari et al., 2018; Ribeiro et al., 2018). MIR is a spectroscopic technique to study the fundamental vibrations and associated rotational-vibrational structure of chemical bonds (Machado et al., 2018; Skolik et al., 2019). MIR is used to identify the chemical components of plants and monitoring the change in those components can help to identify plant growth status. LIBS is a spectroscopic technique to detect elements and their concentrations by analyzing spectral signal constituted by the light emission from laser plasma (Peng et al., 2016). LIBS is used for quantitative and qualitative analysis of elements in plants.

Among these three spectroscopic techniques, HSI (Knauer et al., 2017; Thomas et al., 2018) is the most widely used technique for plant disease detection, while fewer studies have used MIR (Hawkins et al., 2010; Zhang et al., 2017) and LIBS (Ponce et al., 2018; Liu et al., 2019) for disease detection. Thomas et al. adopted HSI to detect barley cultivars inoculation with powdery mildew. An accurate assessment of the disease severity for all six cultivars at measurements over 30 days was achieved (Thomas et al., 2018). Luo et al. (2019) applied HSI to grade the severity of rice blast, and the probabilistic neural network obtained the best performance with the highest classification accuracy of 97.8%. For MIR, Zhang et al. (2017) explored and validated the feasibility of using MIR to detect oilseed rape leaves infected with Sclerotinia stem rot. Healthy and infected leaves had a difference on the average MIR spectra, and the accuracy over 80% was achieved with three chemometric methods. In terms of disease detection with LIBS, Ponce et al. (2018) applied LIBS for discrimination between healthy and Huanglongbing-affected citrus. The wavebands that had the most obvious difference between healthy and HLB-affected trees were the same for all species. With chemometric analysis, the healthy status of plants was differentiated with a high degree of precision.

Several groups of scientists are involved in disease detection using spectral features and modeling (Alberto, 2018; Luo et al., 2019; Gaoqiang et al., 2020). However, these studies did not detect different diseases simultaneously. In natural conditions, there are various diseases affecting rice growth, and they can happen within one field. Since many external and internal symptoms of rice leaves are similar under different disease pressures, it is difficult to detect multiple diseases simultaneously. For this reason, it is of significant importance and applicability to detect various rice diseases at the same time with one model. Besides, only one detection technique was used to detect plant diseases in each study. Although the single dataset can be used to solve the same problem, a combination of information from different modalities have the potential to provide a better understanding of the problem since each technique has unique advantages as well as limitations.

Information fusion of multiple modalities is the key to combine these three techniques. However, the use of datasets with various modalities is a challenging issue. In general, information fusion can be categorized as low-level fusion (raw data are directly combined), mid-level fusion (features extracted from the raw datasets are combined), and high-level fusion (known as decision fusion, the decision results are combined) (Castanedo, 2013; Borras et al., 2015; Zhou et al., 2020). Information fusion aims to reveal the benefits of multisensor measurement, and they are expected to perform better than individual sensors, providing more robust and accurate decisions. In this study, the three fusion levels of HSI, MIR, and LIBS to detect rice diseases were explored.

The objective of this study was to use HSI, MIR, and LIBS to detect three different diseases of rice, including rice leaf blight, rice blast, and rice sheath blight. The specific objectives were to (1) explore the spectral differences among rice leaves inoculated by different diseases; (2) conduct low-level, medium-level, and high-level data fusion for disease identification; (3) develop detection models based on fused data and nonfused data.

## Materials and Methods

### Sample Preparation

To verify the proposed methods in this article being effective despite rice varieties, two different rice varieties were used in this study, including a commercial variety (Zhefujing83) and a newly developed variety (AD516, which is provided and cultivated by the Institute of Crop Science and Nuclear Technology Utilization, Zhejiang Academy of Agricultural Sciences, Hangzhou, China). After 1 month of sowing seed into the seed plots, the seedlings were transplanted into the laboratory greenhouse with regular fertilization and watering.

To obtain inoculated samples, the *in vitro* inoculation method was applied. Rice blast and rice sheath blight are fungal diseases, while rice leaf blight is a bacterial disease. Thus, funguses of rice blast and rice sheath blight were cultured on potato dextrose agar medium, and bacteria of rice leaf blight were cultured in conical flasks.

The leaves cut from healthy plants were used to inoculate fungus and bacteria. Then the leaves were put into sterilized plastic flat plates. To prevent the rice leaves from drying out, the leaves were placed on distilled water–sterilized wipes. For rice blast and rice sheath blight inoculation, the mycelial pellets were placed on the leaves, with two or three pellets per leaf. For rice leaf blight inoculation, the solutions of bacteria were sprayed on to the leaf surface. After inoculation, the plates were sealed and then placed in a room with a temperature of about 26°C and relative humidity about 60%, and healthy leaves were used as control. Four days later, leaves with visible symptoms were collected.

Representative images of diseased leaves are shown in Supplementary Figure 1. The infected leaves were collected for hyperspectral image acquisition. The number of leaves used in this study is presented in Table 1. Six or twelve leaves were acquired in one image. If an image contained 12 leaves, this image would be divided into two subimages with six leaves in each image. After hyperspectral image acquisition, the six leaves were dried as one sample for MIR and LIBS analysis in an oven at the temperature 75°C for 12 h a day for 3 days in a row. Then the dried leaves were placed into centrifuge tubes and ground into powder using an electrical grinder for 5 min with an oscillation frequency of 60 Hz. Unground leaf veins were removed from the centrifuge tubes.

**TABLE 1:** The number of leaves under different disease inoculations (six leaves per sample).

Cultivar	BYK^*a*^	DWB^*b*^	WKB^*c*^	CK^*d*^
Zhefujing83	276 (46)^*e*^	324 (54)	312 (52)	240 (40)
AD516	294 (49)	330 (55)	336 (56)	264 (44)

*The numbers in the brackets were the numbers of samples to be studied. The numbers outside the brackets were the numbers of leaves. ^*a*^The samples inoculated by rice leaf blight. ^*b*^The samples inoculated by rice blast. ^*c*^The samples inoculated by rice leaf blight. ^*d*^The samples inoculated by rice sheath blight. ^*e*^Six leaves were used as one sample.*

Regarding data splitting, 30 and 5 samples of each category were randomly selected into the training set and validation set, and the remaining samples of each category were all selected into an external test set. Besides, the order of samples in different sets of three spectra was the same. In this study, the category value of the healthy samples (CK) was assigned as 0, and the category values of the samples inoculated by rice leaf blight (BYK), rice blast (DWB), and rice sheath blight (WKB) were assigned as 1, 2, and 3, respectively.

### Hyperspectral Image Acquisition and Spectra Extraction

A visible/near-infrared hyperspectral imaging system covering the spectral range of 400–1,000 nm was used to acquire hyperspectral images of healthy and infected leaves. The hyperspectral imaging system is formed by an imaging spectrograph (ImSpector V10E; Spectral Imaging Ltd., Oulu, Finland), a highly sensitive EMCCD camera (Raptor EM285CL, Raptor Photonics limited, Larne, United Kingdom), and a long camera lens (OLES23; Specim, Spectral Imaging Ltd., Oulu, Finland). The illumination of the system is provided by 150 W tungsten halogen lamps (3900 Lightsource, Illumination Technologies Inc., United States). This hyperspectral imaging system conducts line scanning, and a moving plate driven by a stepper motor (GYB751D5-RC2, Fuji Electric (Dalian) Co., Ltd., Dalian, China) is used to move the samples.

To acquire clear and nondeformable images, the distance between the camera lens and the moving plate, the exposure time of the camera, and the moving speed of the moving plate was adjusted to 26 cm, 55 ms, and 2.6 mm/s. The acquired hyperspectral images were then corrected by using the white reference image (acquired using a piece of pure white Teflon board with nearly 100% reflectance) and the dark reference image (acquired covering the lens using a black lens cap with nearly 0% reflectance) according to the following equation:

(1) $$I_{C}=\frac{I_{R}-I_{D}}{I_{W}-I_{D}}$$

where *I*_*C*_ is the corrected image, *I*_*R*_ is the raw image, *I*_*W*_ is the white reference image, and *I*_*D*_ is the dark reference image.

After image correction, all six leaves were defined as a region of interest (ROI); wavelet transform (Wavelet function: Daubechies 8; Decomposition level: 3) was used to denoise the pixel-wise spectra. The average spectrum of the six leaves was calculated as one sample spectrum. The head and the tail of the spectra contained obvious noises produced by the hyperspectral imaging system. Only the spectra in the range of 448–945 nm were used for analysis.

### Mid-Infrared Spectra Collection

To conduct MIR spectra acquisition, rice leaves were dried, ground, and pressed into pellets. Potassium bromide (KBr) was used to be mixed with the leaf powders for mid-infrared spectra acquisition. The KBr powders were first dried at 105°C in an oven for 4 h and then mixed. To obtain the pellet, 0.02 g leaf powders and 0.98 g KBr powders were weighed and mixed thoroughly. Then 0.2 g mixtures were used for tableting. To obtain mid-infrared spectra, a Nicolet iS10 FT-IR spectrometer (Thermo Fisher Scientific^TM^, Madison, WI, United States) was used. The spectral range was 400–4,000 cm^–1^, and the spectral resolution was set as 4 cm^–1^. For each sample, 32 measurements were conducted, and the average value was used as the transmittance spectrum of the sample to reduce variations and random noises. Before spectra acquisition, background correction was performed, and the background correction was conducted every 30 min during spectra acquisition. The spectral data was saved as .csv format for further analysis.

### LIBS Spectra Acquisition

To conduct LIBS spectra acquisition, 0.1 g rice leaf powders were used for tableting. The tablets were dried for 4 h at 75°C before LIBS spectra acquisition. An assembled LIBS system was used to acquire LIBS spectra. The LIBS system used in this research consists of a Q-switch Nd:YAG nanosecond pulsed laser (Vlite-200, Beamtech, Beijing, China). The second harmonic laser (532 nm, pulse duration of 8 ns, beam diameter of 7 mm) was used to ignite the sample with the help of a plano-convex lens (*f* = 50 mm). The detection system consists of a Mecchelle spectrograph (ME5000, Andor, Belfast, United Kingdom) and an ICCD camera (DH334, Andor, Belfast, United Kingdom), which was used to collect plasma emission spectra with the range from 230 to 880 nm. Samples were placed at an X-Y-Z translation stage.

To improve the signal to noise rate, the delay time and the integration time were optimized to 1.5–10 μs, respectively. The laser was fired with a pulse energy of 60 mJ at 1 Hz. For each sample, 10 successive spectra were acquired at each location, and 16 different locations were measured with the help of translation stage in ambient air.

### Data Feature Extraction

Since full spectra of HSI, MIR, and LIBS have high dimensionalities of features, it will increase computing time and the difficulty to build models. Reducing the feature dimensions while keeping the most useful information is a good approach to make full use of the features. Feature extraction methods are effective tools to extract most informative features for dimension reduction. In this study, two feature extraction methods used in spectral data analysis were applied for feature extraction, principal component analysis (PCA), and autoencoder (AE).

PCA is a widely used feature extraction method for data compression in spectral data analysis. It can reduce the dimensionality of spectral information through calculating the linear combination of the original data (Diniz et al., 2014). The variables after transformation are called principal components (PCs). After the orthogonal transformation of PCA, the first few PCs contain a majority of the information pertaining to the original variables (Zhu S.et al., 2019). The accumulative explained variance determines the number of PCs. Considering making use of original information as much as possible, the first few PCs with accumulative explained variance over 99.99% were adopted as extracted features in this study. Therefore, the number of PCs of three different sources was not identical.

The deep learning (DL) framework has been introduced into feature extraction and data reduction in spectra analysis recently due to its powerful representation ability. As part of the DL framework, the AE network can learn abstract features through the hidden layer in an unsupervised manner (Zabalza et al., 2016), which makes AE quite popular for feature extraction. With the assistant of hidden layers in neural networks, data reduction is achieved while maintaining the effective information of the data (Zabalza et al., 2016).

**FIGURE 1:**
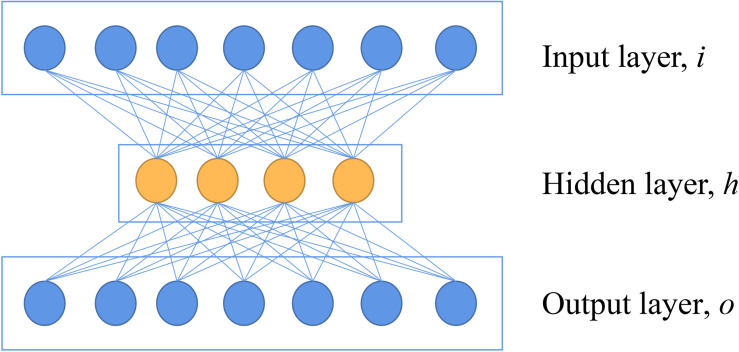
The basic architecture of autoencoder.

AE is a neural network that reconstructs the value of output to be as the same as possible to the value of the input, which indicates the output layer has the same number of nodes as the input layer (Xing et al., 2016). A basic architecture of AE can be seen in Figure 1. In the encoder part, a basic AE has an input layer of *i* neurons, which is equal to the dimensionalities of features of the input. A hidden layer with *h* neurons (*h* < *i*) following with the input layer is also introduced. This hidden layer is used to extract features with an activation function. In the decoder part, the *h* is mapped to an output layer with *o* neurons (*o* = *i*) to reconstruct the input data (Lin et al., 2013). This network is used to reconstruct the original spectra by minimizing the loss of mean squares, which maintains the key information of original data.

AE can simply introduce several hidden layers between the input and the output for feature extraction. After several trials, the shallow AE network was found to be more effective for reconstructing the original spectra of rice leaves. The architectures of AE used in this work and the change of data dimensionalities are reported in Supplementary Figure 2. For HSI, the input layer was a fully connected layer, and dimensionalities of the output were 64. Next, the dimensionality of data became 32 after encoder, and the features were further used for classification. Then the decoder increased the dimensionality of data to 64, and further increased to the same dimensionality of input data. Apart from the last output layer in the AE, the other three fully connected layers were all followed by a batchnorm layer. Besides, all other three layers used the rectified linear unit (ReLU) as an activation function while the last output layer did not use any activation function. The data dimensionalities from beginning to the end could be simply recorded as 390–64–32–64–390. In terms of MIR spectra, the architecture with total four layers was also adopted, and the change of dimensionalities could be simply recorded as 7,468–64–16–64–7,468. Since LIBS spectra had over 20,000 dimensionalities of features, we increased the dimension of the output of the first layer. Thus, the change of dimensionalities could be simply recorded as 22,036–256–64–256–22,036.

### Data Fusion Strategies

Data fusion is a method to fuse data collected from multiple sensors (Doeswijk et al., 2011). To fully dig effective information from different sources, different kinds of data fusion strategies were used to investigate the feasibility of combining the information with HSI, MIR, and LIBS datasets for rice disease detection.

#### Low-Level Data Fusion

In the case of low-level fusion strategy, the original HSI, MIR, and LIBS datasets were concatenated into a single matrix. Two or three data sources were fused. The four combinations were listed as follows: HSI-MIR (this means the fusion of HSI spectra and MIR spectra, and other abbreviations are similar to this fashion), HSI-LIBS, MIR-LIBS, and HSI-MIR-LIBS. However, the full use of the datasets of different spectra is a challenge because the informative parameters with small value will be ignored due to the existence of large value parameters during data fusion (Wang et al., 2016). Therefore, a z-score normalization was applied firstly to rescale the spectra values of different sensors before the model construction.

#### Mid-Level Fusion

**FIGURE 2:**
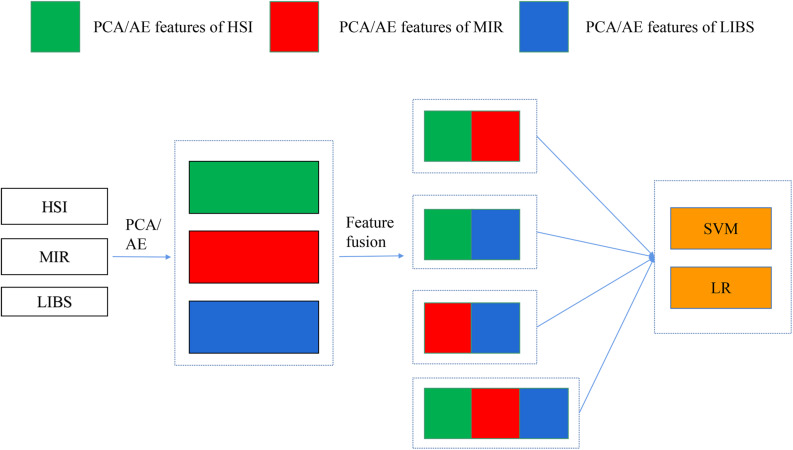
Concatenation of PCA/AE features of different spectra.

**FIGURE 3:**
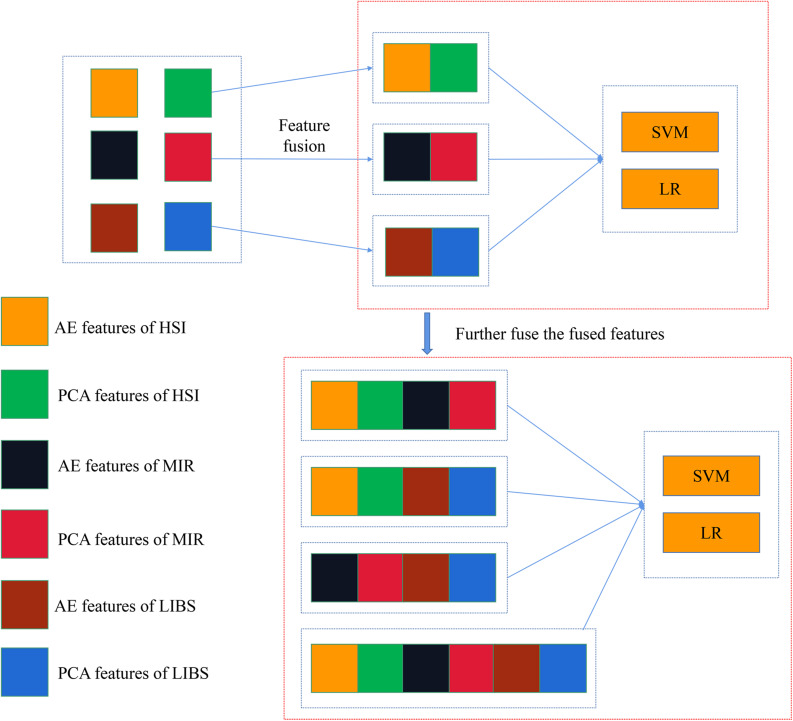
Concatenation of AE features and PCA features of the same spectra and further concatenating the fusion data of different spectra.

In mid-level fusion, two strategies were adopted: (1) concatenating the PCA/AE features from three data sources, respectively. As shown in Figure 2, PCA features separately extracted from three kinds of spectra were concatenated. There were four combinations for PCA features: PCA-HSI-MIR (this means concatenating the PCs of HSI and the PCs of MIR, and other abbreviations are similar to this fashion), PCA-HSI-LIBS, PCA-MIR-LIBS, and PCA-HSI-MIR-LIBS. In terms of AE features, fused data could be briefly recorded as AE-HSI-MIR, AE-HSI-LIBS, AE-MIR-LIBS, and AE-HSI-MIR-LIBS; (2) concatenating the PCA features and the AE features from the same data source, and the fused data can be briefly recorded as AE-PCA-HSI, AE-PCA-MIR, and AE-PCA-LIBS. Furthermore, the aforementioned concatenated features of three different instruments were further combined. These combined features could be briefly recorded as AE-PCA-HSI-MIR, AE-PCA-HSI-LIBS, AE-PCA-MIR-LIBS, and AE-PCA-HSI-MIR-LIBS, as shown in Figure 3.

#### High-Level Fusion

The high-level fusion is also called decision-level fusion. Majority voting is commonly applied to make the final prediction according to the prediction results of single models (Ballabio et al., 2018). For the same modeling method, models were built using HSI, MIR, and LIBS datasets, and the voting weights of each dataset were set as equivalent. For the same sample, if two or three of the models predicted it as the same value, then this value was set as the final prediction value of the sample. If the prediction value of the same sample by the models using the three datasets were all different, it means that the sample was unidentified.

In summary, we conducted three fusion strategies for rice disease detection: (1) the preprocessed spectra were concatenated as a new dataset to build classification models directly; (2) to conduct mid-level fusion, PCA and AE were used as feature extraction methods. The features extracted by the same feature extraction method (PCA/AE) of the three datasets were combined. Also, the features extracted by different feature extraction methods of each dataset were combined. Furthermore, aforementioned fused features of the three datasets were further combined; (3) decision fusion was conducted by combining the prediction results of different datasets using the same modeling method.

### Classification Methods

Discriminant methods were used in this study to classify different rice diseases. These methods included logistic regression (LR), support vector machine (SVM), and convolutional neural network (CNN).

LR is a linear regression method used for classification. LR model is basically used to solve the binary classification problems. It outputs probabilities of two situations, and the class of the corresponding sample is determined based on the probabilities. The general idea of LR is to map the real value predicted by the linear regression model into the value in range 0–1 (probability) by a sigmoid function. LR can also be extended to multiclass classification problems by using the one vs. rest strategy (Tremblay et al., 2019; Zhu S.S.et al., 2019).

SVM is a widely used machine learning method for classification and regression. For linearly separable data, a linear equation can be obtained to construct the hyperplanes. For data which are not linearly separable, SVM maps the original data into high-dimensional spaces to transform the problem into linearly separable issues and constructs hyperplanes to maximally divide the samples from different categories in the new spaces (Feng et al., 2018; Sun et al., 2018; Zhang et al., 2018). Kernel functions are essential for the mapping, and radial basis function (RBF) is a widely used kernel function of SVM. In this study, RBF was used as the kernel function for SVM with a grid-search procedure for parameter optimization.

CNN is a promising method in various fields nowadays. CNN consists of multiple convolution layers and pooling layers, which enable this neural network to extract abstract shallow and deep features of the input automatically. Due to its powerful representative ability, CNN recently has been introduced to vibration spectral data analysis for classification (Wu et al., 2018; Feng et al., 2019; Zhu S.S.et al., 2019) and regression (Ng et al., 2020; Zhang et al., 2020).

**FIGURE 4:**
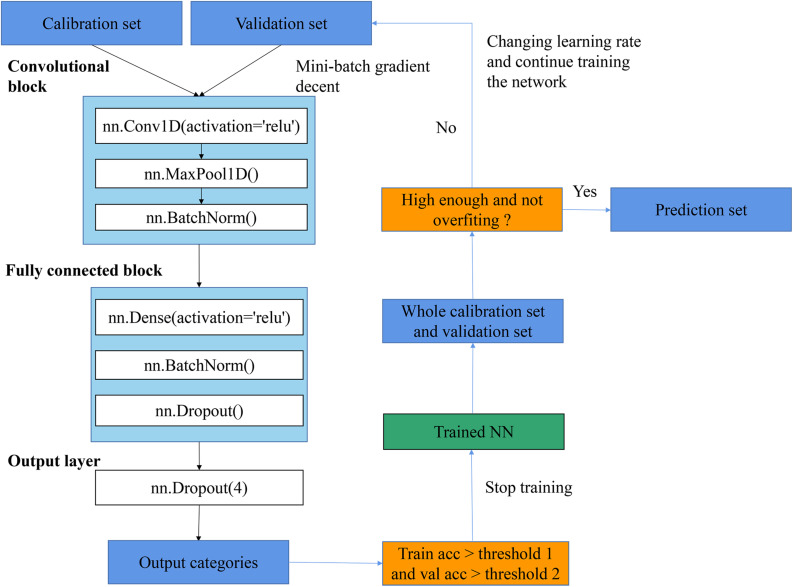
The flowchart of procedures of training CNN.

As shown in Figure 4, the general flowchart of training CNN in this article included: (1) feeding training set and validation set into a network and keep training with changing learning rate until the training accuracy and validation accuracy reach up to thresholds; (2) applying the trained network to predict the whole training set and validation set and inspecting whether the training accuracy and validation accuracy were high enough and whether the overfitting problem existed; (3) if the accuracy was high enough and the overfitting did not exist, the trained network was saved and all results with this trained network were recorded; otherwise, changing the learning rate and keep training the network. The training procedure was performed by optimizing the cross-entropy loss with the SGD algorithm. Furthermore, the relationship between epochs and training performances is provided in Supplementary Figure 3, which illustrated the change of training accuracy and train loss as the change of epochs. This is an initial step of training process. After this step, several small learning rates were used to finetune the CNN, and a threshold (e.g., accuracy >0.98) was set to stop the training process (which is not shown in the figure).

### Model Establishment, Evaluation, and Software

To develop the SVM model, the RBF kernel was set as the kernel function. The optimal combination of penalty parameter (*c*) and kernel coefficient parameter gamma (*γ*) of SVM was searched using the GridSearchCV function provided by scikit-learn (version 0.21.3), a Python machine learning package. The search range of parameters was set as 10^–10^ to 10^10^ for both *c* and *γ*, and the three-fold was used for cross-validation strategy. In addition, the scoring parameter of the function was set as “accuracy.” In terms of the optimization algorithm of LR, “liblinear” was chosen for L1 penalization and “newton-cg” was chosen for L2 penalization in this article. After optimizing these parameters, a fine-tuning of the parameters was implemented on the training set and the validation set. Except for the traditional SVM and LR models, CNN was also used for the classification task. Since the dimensionalities of features of raw spectra were varied with the type of technique, the architectures of CNN were not identical. Besides, different spectra data have different data structures, and different CNN architectures were tried and a superior one was chosen for each specific input to get better results (shown in Supplementary Figures 4–19).

Each preprocessed spectrum of each technique was further implemented with the standardization process before being fed into SVM and LR. This standardization preprocessing standardized features of the training set by removing the mean and scaling to unit variance and performed the same standardization on the validation set and the test set by a utility class StandardScaler in scikit-learn. Concerning modeling with PCA features, the standard preprocessing was also applied. According to self-designed AE, 32 dimensionalities features of HSI spectra extracted by AE were fed into SVM and LR models. Before being fed into SVM and LR, features of the training set, the validation set, and the test set were firstly standardized by the StandardScaler as mentioned before. This standard preprocessing was also applied to AE features of MIR and LIBS. In terms of building CNN, data without the preprocessing of StandardScaler was found to be helpful to achieve better performance. Therefore, each source of spectra, PCA features, and AE features were directly fed into CNN. In terms of features of different levels of fusion, Standardscaler transformation was applied on merged datasets to compensate for the scale differences before feeding these features into classifiers.

The spectra extraction of HSI, MIR, and LIBS were conducted on MATLAB R2015b (The Math Works, Natick, MA, United States). To evaluate the model performances, classification accuracy was used, which was the ratio of the correctly classified number of samples and the total sample number. Deep learning was conducted by python3 with MXNET framework (Amazon, Seattle, WA, United States) with GPU acceleration. A computer with Intel Core-i7 8700k CPU, NVidia GTX1060 GPU, 16 GB RAM, and 256 GB SSD was used for calculation.

## Results

### Spectral Profiles

**FIGURE 5:**
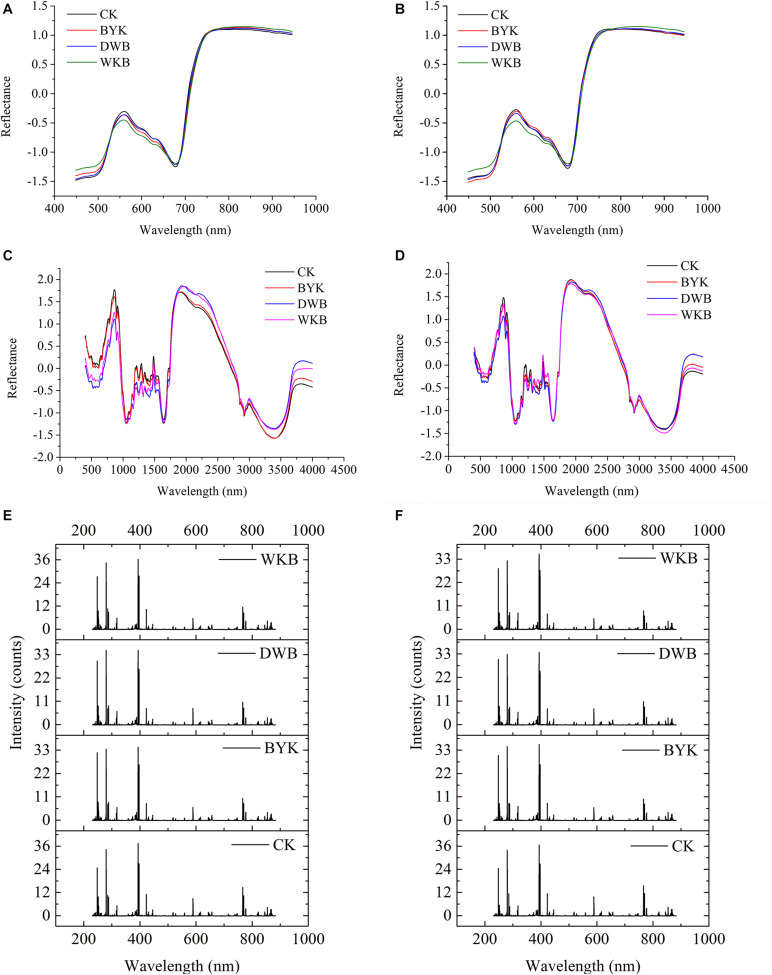
The average spectra of two varieties of rice leaves in different health conditions. **(A)** HSI spectra of Zhefujing83; **(B)** HSI spectra of AD516; **(C)** MIR spectra of Zhefujing83; **(D)** MIR spectra of AD516; **(E)** LIBS spectra of Zhefujing83; **(F)** LIBS spectra of AD516.

After standardization preprocessing for each dataset, the average spectra for both rice cultivars were plotted for visualization. Figures 5A,B shows the average HSI spectra of healthy and infected leaves of the two rice cultivars. Figures 5C,D presents the average MIR spectra of healthy and infected leaves of the two rice cultivars. Figures 5E,F shows the average LIBS spectra of healthy and infected leaves of the two rice cultivars. The differences between HSI spectra, MIR spectra, and LIBS spectra could be observed intuitively.

Concerning HSI spectra, there was some noise at the beginning and end of spectra due to the response of the spectroscopic instrument, which made a difference among curves of leaves in four health conditions. Therefore, this range of spectra is out of the discussion. The spectra among healthy leaves and leaves suffering from three different diseases presented an obvious difference in the range of 530–650 nm. The average spectra of healthy leaves were distinguishingly higher than leaves of DWB and WKB around the spectral region of 530–650 nm. However, some overlaps existed among the spectral curves of leaves under different conditions. Therefore, it required further study to make a better distinction.

There were obvious differences among the MIR spectra of leaves in four different health conditions as well. In the range of 500–1,500 cm^–1^, the spectra of healthy leaves of the Zhefujing83 had higher values than the leaves infected with DWB and BYK. On the contrary, the spectra of healthy rice leaves of the Zhefujing83 had lower values than the leaves infected with DWB and BYK around 2,000–2,750 nm. In terms of the AD516, the tendency of MIR spectra was similar.

As for LIBS spectra, each peak of LIBS spectra was related to the specific element, which would vary with the change of environmental and intrinsic factors (such as the health condition). With respect to Zhefujing83, the peak intensity of leaves infected by DWB and BYK were smaller than that of healthy leaves. In terms of AD516, the peak intensity of leaves with WKB, DWB and BYK were all smaller than that of healthy leaves.

### Classification Models Using Each Single Spectroscopic Datasets

The results of the classification models using each single spectroscopic datasets are shown in Table 2. Among the three original spectroscopic datasets, the highest prediction accuracy was obtained from HSI spectral data of two rice varieties. CNN model with HSI data of Zhefujing83 could achieve the accuracy of 100% for the test set that was better than 90.38% (SVM) and 98.08% (LR). As for AD516, both CNN and LR models using HSI data achieved the accuracy of 100% for the training set, the validation set, and the test set. Furthermore, the confusion matrix of CNN based on the full HSI spectra of AD516 is illustrated in Supplementary Figure 20. The confusion matrix indicated the three diseases were separable based on the spectral data.

**TABLE 2:** The classification results based on multisource spectra.

Cultivar	Features	Variables	Model	Tr (%)	Val (%)	Te (%)	Model	Tr (%)	Val (%)	Te (%)	Model	Tr (%)	Val (%)	Te (%)
Zhefujing83	Full-HSI	390	SVM	95.00	90.00	90.38	LR	98.33	95.00	98.08	CNN	**98.33**	**95.00**	**100**
	Full-MIR	7,468		100	100	76.92		**100**	**100**	**82.69**		100	95.00	**82.69**
	Full-LIBS	22,036		100	100	71.15		100	100	67.31		**100**	**100**	**86.54**
AD516	Full-HSI	390		100	100	96.88		**100**	**100**	**100**		**100**	**100**	**100**
	Full-MIR	7,468		99.17	90.00	85.94		**100**	**100**	**96.88**		100	90.00	81.25
	Full-LIBS	22,036		100	100	75.00		**100**	**100**	**87.50**		100	100	81.25

*Other abbreviations are similar to this fashion. Tr, training set; Val, validation set; Te, test set; Full-HSI, full spectra of HIS.*

In terms of the results of MIR, both CNN and LR using MIR data of Zhefujing83 obtained the accuracy of 82.69% for the test set, which was higher than SVM (79.69%). As for AD516, LR obtained the best performance, with the accuracy of 96.88% for the test set.

Based on Full-LIBS spectra of Zhefujing83, CNN (the accuracy of 86.54% for the test set) obtained a higher accuracy of 15.39 and 19.23% for the test set than the corresponding SVM (71.15%) and LR (67.31%), respectively. CNN with Full-LIBS spectra of AD516 were inferior to the corresponding LR but superior to the corresponding SVM. This might result from the severity of diseases varying with rice variety.

### Classification Models Based on Feature Extraction

#### PCA-Based Classification

The results of models based on features extracted by PCA are shown in Table 3. Among PCA features from three spectroscopic instruments, the highest prediction accuracy was obtained from PCA features of HSI spectra for both rice varieties.

**TABLE 3:** The classification results based on PCA/AE features of different spectral sources.

Cultivar	Features	Variables	Model	Tr (%)	Val (%)	Te (%)	Model	Tr (%)	Val (%)	Te (%)	Model	Tr (%)	Val (%)	Te (%)
Zhefujing83	PCA-HSI	14	SVM	98.33	95.00	100	LR	**98.33**	**100**	**100**	CNN	100	95.00	94.23
	PCA-MIR	34		**100**	**100**	**90.38**		**100**	**100**	**90.38**		100	95.00	90.38
	PCA-LIBS	114		100	85.00	63.46		100	85.00	63.46		**100**	**95.00**	**78.85**
	AE-HSI	32		**98.33**	**70.00**	**76.92**		98.33	70.00	65.38		98.33	80.00	63.4
	AE-MIR	16		**94.17**	**80.00**	**84.38**		96.67	95.00	76.92		100	100	78.85
	AE-LIBS	64		99.17	95.00	63.46		99.17	90.00	65.38		**100**	**100**	**69.23**
AD516	PCA-HSI	14		100	100	96.88		**100**	**100**	**100**		100	100	93.75
	PCA-MIR	42		**100**	**100**	**96.88**		100	100	95.31		100	95.00	78.13
	PCA-LIBS	103		100	90.00	81.25		100	95.00	81.25		**95.83**	**95.00**	**85.94**
	AE-HSI	32		94.17	75.00	81.25		**97.5**	**95.00**	**84.38**		95.00	85.00	84.38
	AE-MIR	16		**96.67**	**80.00**	**87.50**		95.00	85.00	84.38		99.17	85.00	78.13
	AE-LIBS	64		100	90.00	73.44		**100**	**95.00**	**81.25**		100	95.00	73.44

*Other abbreviations are similar to this fashion. Tr, training set; Val, validation set; Te, test set; PCA-HSI, the features of HSI extracted by PCA; AE-HSI, the features of HSI extracted by AE.*

In terms of HSI, the first 14 PCs explaining 99.99% of the information in original spectra were adopted to modeling. For two rice varieties, the accuracy of the training set of SVM and LR models were all over 98%, while the accuracy of the validation set and the test set exceeded 96%. CNN with PCA features of HSI spectra obtained the accuracy over 93% for both varieties. Overall, models based on PCA features achieved better performance than models based on full spectra.

With respect to MIR, the number of PCs for two rice varieties was not identical in the condition of the same accumulative explained variance. The results of the training set and validation set all reached 100% based on both SVM and LR models, and the results of the test set were all above 90%. CNN obtained the accuracy of 90.38% for the test set as well. Compared with the results based on Full-MIR spectra, the performance of the three models was improved overall. This indicted PCA was not only helpful in reducing the dimension of data but also contributed to improving classification accuracy.

As for LIBS, the number of PCs for two rice varieties was also not identical for a similar reason. After parameter optimization, classification results of SVM and LR for the training set were all 100% for both varieties, and the prediction results of AD516 were 81.25%, while prediction results of the Zhefujing83 were 63.46%. These results revealed that the variety of rice might have an influence on rice leaf disease discrimination. CNN model based on PCA-LIBS of Zhefujing83 obtained the accuracy of 100 and 95% for the training set and the validation set, respectively, which were better than corresponding SVM and LR models. The accuracy for the test set increased from 63.46% (SVM and LR) to 78.85% (CNN). The change trend of results of CNN for the AD516 was similar to the Zhefujing83.

Compared with the results based on Full-HSI spectra, the accuracy of models based on PCA features was improved but with only 14 dimensionalities of features. This contributed to saving computing resources and computing time. In addition, the results based on PCA features of Full-MIR data were obviously improved than the results based on Full-MIR data, while the dimensionality of data was decreased from 7,468 to 34 for Zhefujing83 and 42 for AD516. The results based on Full-LIBS data was overall slightly better than the results based on PCA-LIBS. However, the models with PCA-LIBS only used less than 114 dimensionalities (114 for Zhefujing83 and 103 for AD516) of data, while the former used 22,036 dimensionalities of Full-LIBS data. Overall, PCA was an effective tool for feature extraction.

#### Autoencoder-Based Classification

As shown in Table 3, the accuracy of the test sets of two rice varieties all exceeded 75% by SVM models using AE features. Meanwhile, LR only achieved the accuracy of 84.38% for the test set of AD516 but obtained only 63.58% for Zhefujing83. CNN based on AE-HSI obtained the accuracy of 67.31% for the test set of Zhefujing83 and obtained 79.69% for AD516.

In terms of AE-MIR, the performance of the SVM model was superior to the LR model after parameter optimization, with the accuracy surpassing 94% for the training set, reaching 80% for the validation set, and all the accuracy over 84% for the test set. Compared with the results based on Full-MIR spectra, SVM based on features from AE performed slightly better. Meanwhile, LR based on AE-MIR obtained worse classification results than LR based on Full-MIR. For Zhefujing83, CNN based on AE-MIR obtained the accuracy of 100% for both the training set and the validation set. However, it only obtained 75% for the test set. Besides, CNN obtained 78.13% for the test set of AD516. It indicated CNN had limited power on the small dataset (only 30 samples per category of rice). Besides, the results based on AE were inferior to the results based on PCA. The features extracted from AE had no more than half of the dimensionalities of features extracted by PCA.

Considering the massive dimensionalities of the Full-LIBS spectra, 64 dimensionalities of features were extracted for classification models. The performance of SVM and LR models using AE-LIBS were similar, and both models obtained the accuracy surpassing 99% for the training set and the accuracy surpassing 90% for the validation set for both rice varieties. CNN (71.88% for the test set) based on AE-LIBS was inferior to the corresponding SVM (73.44% for the test set) and LR (81.25% for the test set). Besides, all three classifiers obtained a higher accuracy for AD516 than Zhefujing83. It indicated that the variety of rice influenced feature extraction.

Overall, the performance of models varied with the feature extraction methods. This might attribute to that different feature extraction methods learned and represented different aspects of the features of the original data. Integrating features extracted by different methods might contribute to improving classification performance. Thus, feature fusion was conducted for classification.

### Classification Models Based on Data Fusion

#### Low-Level Fusion

In the low-level fusion approach, spectra from different sources were directly concatenated. After parameter optimization, the results of SVM, LR, and CNN are presented in Table 4.

**TABLE 4:** The accuracy rates of classification models based on fusion data.

Cultivar	Features	Variables	Model	Tr (%)	Val (%)	Te (%)	Model	Tr (%)	Val (%)	Te (%)	Model	Tr (%)	Val (%)	Te (%)
Zhefujing83	HSI-MIR	7,858	SVM	100	95.00	84.62	LR	**100**	**100**	**86.54**	CNN	100	95.00	84.62
	HSI-LIBS	22,426		100	100	76.92		100	100	69.23		**100**	**100**	**90.38**
	MIR-LIBS	29,504		**100**	**100**	**86.54**		100	100	80.77		**100**	**100**	**86.54**
	HSI-MIR-LIBS	29,894		100	100	90.38		100	100	82.69		**100**	**100**	**92.31**
AD516	HSI-MIR	7,858		100	100	92.19		**100**	**100**	**100**		100	95.00	85.94
	HSI-LIBS	22,426		100	100	81.25		**100**	**100**	**90.63**		100	100	84.38
	MIR-LIBS	29,504		100	100	84.38		**100**	**100**	**92.19**		100	100	84.38
	HSI-MIR-LIBS	29,894		100	100	85.94		**100**	**100**	**92.19**		100	100	81.25

*Other abbreviations are similar to this fashion. Tr, training set; Val, validation set; Te, test set; HSI-MIR, the concatenation of full HSI and MIR spectra; HSI-LIBS, the concatenation of full HSI and LIBS spectra; MIR-LIBS, the concatenation of full MIR and LIBS spectra; HSI-MIR-LIBS, the concatenation of full HSI, MIR, and LIBS spectra.*

The combination of HSI and MIR helped to improve classification accuracy by about 4–6% compared with Full-MIR spectra. Moreover, models based on HSI-LIBS fusion data obtained better performance than models based on the Full-LIBS spectra. However, the accuracy based on HSI-MIR and HSI-LIBS declined compared with the results only based on the HSI data. The reason might attribute to the irrelevant information contained in full MIR and LIBS spectra. Fused data of individual spectra had a higher dimension and contained more irrelevant information, which interfered with the discriminative power of classifiers. In addition, compared with Full-MIR, the combination of Full-MIR and Full-LIBS only helped to improve the accuracy of SVM for the Zhefujing83. In contrast, the accuracy of SVM for AD516 and the accuracy of LR models for both rice varieties had all slightly declined.

In terms of Zhefujing83, CNN based on HSI-LIBS achieved an accuracy of 90.38% that was higher than 76.92% (SVM) and 69.23% (LR). For MIR-LIBS, CNN obtained an accuracy of 86.54% for the test set that was around 6% higher than the corresponding LR. CNN based on MIR-LIBS (with the accuracy of 86.54% for the test set) was better than CNN based on Full-MIR alone (with 82.69% for the test set). CNN based on HSI-MIR-LIBS performed better than the corresponding SVM and LR. Besides, CNN based on HSI-MIR-LIBS obtained the accuracy of 92.31% for the test set, which were higher than both CNN based on MIR (82.69%) and CNN based on LIBS (86.54%). In terms of AD516, though CNN achieved the accuracy of 100% for both the training set and the validation set despite the data sources, CNN obtained lower accuracy for the test set when compared with SVM and LR.

In summary, the accuracy based on low-level fusion was not improved, but the dimensionalities of the input data were multiplied. In consequence, the computing resources and computing time were increased. Therefore, the low-level fusion strategy was not efficient enough for classification improvement in this study.

#### Mid-Level Fusion

##### Fusion of AE Features and PCA Features of Each Spectral Dataset

In the case of mid-level fusion, the informative features separately extracted by AE and PCA were concatenated into a single matrix that was further used for multivariate analysis. The results are shown in Table 5. Satisfactory results were obtained with feature fusion analysis.

**TABLE 5:** The classification accuracy based on fusion data of AE and PCA features of different spectral sources.

Cultivar	Features	Variables	Model	Tr (%)	Val (%)	Te (%)	Model	Tr (%)	Val (%)	Te (%)	Model	Tr (%)	Val (%)	Te (%)
Zhefujing83	AE-PCA-HSI	46	SVM	98.33	100	98.08	LR	**98.33**	**100**	**100**	CNN	98.33	85.00	90.38
	AE-PCA-MIR	50		100	100	86.54		**100**	**100**	**88.46**		100	100	86.54
	AE-PCA-LIBS	178		100	90.00	61.54		99.17	95.00	67.31		**100**	**95.00**	**73.08**
	PCA-HSI-MIR	48		100	100	96.15		**100**	**100**	**98.08**		100	95.00	88.46
	PCA-HSI-LIBS	128		100	95.00	96.15		**100**	**100**	**100**		100	85.00	63.46
	PCA-MIR-LIBS	148		**100**	**100**	**86.54**		**100**	**100**	**86.54**		100	95.00	48.08
	PCA-HSI-MIR-LIBS	162		**100**	**100**	**96.15**		**100**	**100**	**96.15**		100	100	80.77
	AE-PCA-HSI-MIR	96		100	100	94.23		**100**	**100**	**98.08**		100	95.00	84.62
	AE-PCA-HSI-LIBS	224		**100**	**95.00**	**75.00**		**100**	**95.00**	**75.00**		**100**	**95.00**	**75.00**
	AE-PCA-MIR-LIBS	228		100	100	88.46		**100**	**100**	**90.38**		100	100	86.54
	AE-PCA-HSI-MIR-LIBS	274		**100**	**100**	**92.31**		**100**	**100**	**92.31**		100	100	78.85
AD516	AE-PCA-HSI	46		100	100	92.19		**100**	**100**	**100**		100	95.00	92.19
	AE -PCA-MIR	58		100	100	95.31		**100**	**100**	**96.88**		100	95.00	84.38
	AE -PCA-LIBS	167		100	85.00	79.69		**100**	**100**	**84.38**		100	95.00	73.44
	PCA-HSI-MIR	56		100	100	98.44		**100**	**100**	**100**		100	95.00	90.63
	PCA-HSI-LIBS	117		**100**	**100**	**98.44**		**100**	**100**	**98.44**		100	95.00	87.5
	PCA-MIR-LIBS	145		**100**	**100**	**85.94**		**100**	**100**	**85.94**		100	95.00	85.94
	PCA-HSI-MIR-LIBS	159		100	100	95.31		**100**	**100**	**98.44**		100	100	87.5
	AE-PCA-HSI-MIR	104		100	100	98.44		**100**	**100**	**100**		100	100	85.94
	AE-PCA-HSI-LIBS	213		100	100	82.81		**100**	**100**	**90.63**		100	95.00	73.44
	AE-PCA-MIR-LIBS	225		100	100	79.69		**100**	**100**	**85.94**		100	100	78.13
	AE-PCA-HSI-MIR-LIBS	271		100	100	82.81		**100**	**100**	**100**		100	90.00	78.13

*Other abbreviations are similar to this fashion. Tr, training set; Val, validation set; Te, test set; AE-PCA-HSI, the concatenation of PCA features and AE features of HSI; PCA-HSI-MIR, the concatenation of PCA features of HSI and PCA features of MIR; AE-PCA-HSI-MIR, the concatenation of AE-PCA features of HSI and AE-PCA features of MIR.*

In terms of HSI, both the Zhefujing83 and the AD516 have 46 variables after concatenation. With respect to Zhefujing83, in contrast with the model performance of the AE-based model, the accuracy for the training set, the validation set, and the test set was improved after data fusion, which was not much different from the results of the PCA feature-based model. The accuracy of SVM based on AE-PCA-HSI improved to 98.33, 100 and 98.08% for the three datasets (training, validation, and test), respectively, when compared with SVM based on AE-HSI (98.33, 70, and 76.92%). The accuracy of LR based on AE-PCA-HSI improved to 98.33, 100, and 100% for the three datasets, respectively, when compared with LR based on AE-HSI (98.33, 70, and 65.38%). The accuracy of CNN based on AE-PCA-HSI improved to 98.33, 85, and 90.38% for the three datasets, respectively, when compared with CNN based on AE-HSI (99.17, 80, and 67.31%). The changing trend of results was similar for AD516. This indicted combining features extracted by different feature extraction methods was helpful to improve classification performance.

In the case of MIR, the performance of models based on this fusion was better than those based on the AE-based model. Concerning Zhefujing83, the results of SVM with AE-PCA-MIR were improved to 100, 100, and 86.54% for the three datasets, respectively, when compared with SVM based on AE-MIR (94.17, 80, and 84.38%). The accuracy of LR with AE-PCA-MIR was improved to 100, 100, and 88.46% for the three datasets, respectively, when compared with LR based on AE-MIR (96.67, 95, and 76.92%). The accuracy of CNN based on AE-PCA-MIR was improved to 100, 100, and 86.54% for the three datasets, respectively, when compared with CNN based on AE-MIR (100, 100, and 75%). The change trend of results was similar with respect to AD516.

In the case of LIBS, compared with the accuracy based on PCA features, the accuracy based on the fusion data were decreased by about 2% for Zhefujing83, which were increased by about 3% for AD516. Overall, integrating features extracted by different feature extraction methods was helpful for better classification performance.

##### Fusion of Features from Three Spectral Datasets

From the section “Classification Models Based on Feature Extraction,” we found that the models based on features extracted from PCA had the best performance compared with models based on the AE features. Thus, the PCA features extracted from three spectroscopic data were further fused for classification. The results are shown in Table 5. SVM and LR were carried out on the fused features using the previously used parameter optimization method.

In terms of the Zhefujing83, on the one hand, in contrast with the results based on PCA-HSI, the accuracy for the training set and the validation set increased to 100% with both SVM and LR models based on the integration of PCA features of HSI and MIR. The accuracy of the test set was 96.15% for SVM and 98.08% for LR. On the other hand, compared with results based on PCA features of MIR, the accuracy for the test set respectively increased by 5.77 and 7.7% for SVM and LR based on integrated features of HSI and MIR.

As for AD516, the models based on PCA-HSI-MIR obtained the best performance among all combinations, followed by PCA-HSI-LIBS, PCA-HSI-MIR-LIBS and PCA-MIR-LIBS. In contrast with the corresponding models using single source of PCA features, the LR based on PCA-HSI-MIR achieved the classification accuracy of 100% for the training set, the validation set, and the test set, which were identical with LR based on PCA-HSI and were better than the LR based on PCA-MIR. Moreover, SVM based on PCA-HSI-MIR obtained good results for the training set, the validation set, and the test set, with the classification accuracy of 100, 100, and 98.44%, respectively. SVM based on PCA-HSI-MIR were better than SVM based on PCA-HSI. Besides, SVM based on PCA-HSI-MIR were better than SVM based on PCA-MIR as well. CNN based on PCA-HSI-MIR achieved 100, 95, and 90.63% for the training set, the validation set, and the test set, respectively.

However, integrating PCA-MIR with PCA-LIBS deteriorated the performance of three kinds of models; the accuracy was 5–10% lower than models based on PCA-MIR. Beyond that, the results based on PCA feature fusion were close to even better than the results based on a single source of PCA features.

Except for integrating PCA features from three kinds of spectra, we further fused the AE-PCA fusion data of different data sources in the previous section. The results are shown in Table 5. Before being fed to SVM and LR, the fusion data were preprocessed by the StandaradScaler as mentioned before.

For Zhefujing83, in contrast with models based on AE-PCA features of MIR, the classification accuracy of the SVM and LR based on AE-PCA-MIR-LIBS was improved by about 2%. The accuracy increased more when compared with models based on the AE-PCA features of LIBS. Besides, SVM and LR based on this kind of fusion had better performance than models based on full spectra overall for both rice varieties. Among all combinations, the performances of models in descending order were as follows: AE-PCA-HSI-MIR, AE-PCA-HSI-MIR-LIBS, AE-PCA-MIR-LIBS, and AE-PCA-HSI-LIBS. CNN obtained the best results using PCA-HSI-MIR, with the accuracy of 100, 95, and 88.46% for the training set, the validation set, and the test set, respectively.

For AD516, in contrast with the best SVM and LR based on the AE-PCA feature from a single data source, the integration of AE-PCA features of HSI and MIR contributed to obtaining better classification results. Besides, the integration of AE-PCA features of three spectra was helpful to obtain the accuracy of 100% for three datasets with LR. CNN based on this kind of fusion did not exhibit good enough performance, though CNN obtained the accuracy of 100% and over 95% for the training set and the validation set, respectively. Among all combinations, CNN using PCA-HSI-MIR obtained the highest accuracy for the test set (90.38%).

In summary, this section studied the feasibility of CNN to classify rice leaves in four conditions with AE-PCA features. The results were inferior to SVM and LR overall. Considering the number of samples in the training set and the validation set being 120 and 20, respectively, CNN may not be able to exhibit its power for classification for lack of enough samples. However, the results could indicate the great potential of deep learning based approaches for rice disease detection. More samples were needed in future studies to fully reveal the advantage of deep learning.

### High-Level Fusion

The high-level fusion strategy based on majority voting was applied to classification results (Tables 2 and 3) obtained by classification models based on a single source of spectra. High-level fusion was applied to full spectra, PCA features, and AE features, respectively. A classifier developed on one specific analytical data made its own predictions. With these predictions from different sources of data, the final decisions of high-level fusion were calculated according to a majority of vote rule. Classification results achieved by using the combination of all three sources of full spectra, the combination of all three sources of PCA features, and the combination of all three sources of AE features are listed in Table 6.

**TABLE 6:** The classification accuracy rate based on high-level fusion.

Feature type	Rice cultivar	Model	Tr (%)	Val (%)	Te (%)	Model	Tr (%)	Val (%)	Te (%)	Model	Tr (%)	Val (%)	Te (%)
Full	Zhufujing83	SVM	100	100	90.38	LR	100	100	88.46	CNN	**100**	**100**	**100**
	AD516		100	100	93.75		**100**	**100**	**98.44**		100	100	87.50
PCA features	Zhufujing83		**100**	**100**	**98.08**		**100**	**100**	**98.08**		100	100	96.15
	AD516		100	100	96.88		**100**	**100**	**98.44**		100	100	90.63
AE features	Zhufujing83		**100**	**100**	**80.77**		99.17	90.00	75.00		100	100	73.08
	AD 516		**100**	**85.00**	**90.63**		100	95.00	89.06		100	90.00	85.94

*Tr, training set; Val, validation set; Te, test set; Full, Full spectra; PCA features, featuers extracted by principal component analysis; AE features, features extracted by autoencoder.*

On the one hand, SVM based on high-level fusion using full spectra of Zhefujing83 obtained the accuracy of 100 and 100% for the training set and the validation set, which were higher than SVM based on Full-HSI spectra (95 and 90%), with the accuracy of 90.38% for the test set of the two SVM models. CNN based on high-level fusion obtained the classification accuracy of 100% for the training set, the validation set, and the test set, which was better than the corresponding CNN based on Full-HSI (98.33, 95, and 100% for the training set, the validation set, and the test set, respectively), Full-MIR (100, 95, and 82.69%) and Full-LIBS (100, 100, and 86.54%). On the other hand, in terms of AD516, the accuracy of SVM for the test set declined to 93.75% after high-level fusion, with the accuracy for the training set and the validation set being the same as 100%. Fusion results of CNN only obtained the accuracy of 87.5% for the test set since both CNN based on Full-MIR and Full-LIBS only achieved 81.25% for the test set. Besides, compared with the accuracy of SVM models based on Full-MIR, the accuracy for the test set was improved from 76.92 to 90.38% for Zhefujing83 and from 85.94 to 93.75% for AD516. When compared with the accuracy of SVM models based on Full-LIBS, the accuracy for the test set was improved by over 18% after high-level fusion.

For high-level fusion of classifiers based on PCA features, the accuracy for both the training set and the validation set was increased to 100% compared with the accuracy based on PCA features of a single type of spectra. In terms of Zhefujing83, SVM based on high-level fusion obtained the accuracy of 98.08% for the test set, which exceeded the corresponding accuracy of SVM based on PCA-MIR and PCA-LIBS. Besides, LR based on high-level fusion obtained the accuracy of 98.08% for the test set, which was much higher than 90.38 and 63.46% of LR based on PCA-MIR and PCA-LIBS, respectively. CNN based on high-level fusion obtained the accuracy of 96.15% for the test set, which were much higher than 94.23, 90.38, and 78.13% of CNN based on PCA-HSI, PCA-MIR, and PCA-LIBS, respectively. With respect to AD516, SVM based on high-level fusion obtained an accuracy of 96.88%, which was 15.63% more than the SVM model based on PCA-LIBS. In addition, LR obtained the accuracy of 98.44% for the test sets, which were higher than 95.31 and 81.25% of LR based on PCA-MIR and PCA-LIBS, respectively. Besides, CNN obtained the accuracy of 90.63% for the test set, which was higher than 78.85 and 85.94% of CNN based on PCA-MIR and PCA-LIBS, respectively.

In terms of high-level fusion of classifiers based on AE features, the SVM and LR models after high-level fusion were all better than models based on individual type of spectra. In terms of Zhefujing83, the accuracy of SVM was increased to 100% for both the training set and the validation set after data fusion, and the accuracy of the test set after high-level fusion was 3.85 and 17.31% higher than that base on AE-HSI and AE-LIBS, respectively. Besides, the improvement trend of the accuracy of SVM and LR for AD516 complied with that of Zhefujing83. For Zhefujing83, CNN based on high-level fusion obtained the accuracy of 73.08% for the test set, which were 5.77 and 17.31% higher than CNN based on AE-HSI and AE-LIBS, respectively. For AD516, CNN based on high-level fusion obtained the accuracy of 85.94% for the test set, which were improved when compared with CNN based on AE-HSI (79.69% for the test set), CNN based on AE-MIR (78.13% for the test set), and CNN based on AE-LIBS (71.88% for the test set).

In all, classification performances based on the high-level fusion approach were slightly better than those based on one single analytical source.

## Discussion

In this study, two different rice varieties were used to verify the proposed methods in this article being effective despite rice varieties. According to Tables 2 and 4, classification accuracy between Zhefujing83 and AD516 was different in general. Overall, LR and SVM obtained higher prediction accuracy for AD516 than those for Zhefujing83. Besides, CNN models for Zhefujing had a better overall performance than those for AD516. There existed a variance among the performance of different models using different datasets of different varieties of rice. The deep reasons for the varietal variances would be further investigated in future studies with more samples with physiological and biochemical analyses.

In terms of data fusion strategies, low-level fusion directly integrates the original data, so it has an immense data calculation. In this study, the original HSI, MIR, and LIBS spectra had 390, 7,468, and 22,036 dimensionalities of features, respectively. After concatenating every two of them, the dimensionalities of new data would greatly increase. This would increase the computing time. Apart from the high dimension of input, the limited number of samples in the training set (only 30 samples per category) restricted the performance of CNN.

Besides, the increase in information brought by low-level fusion may not compensate for irrelevant or spurious variance brought by this fusion strategy (Biancolillo et al., 2014). The low-level fusion has some limitations which are a high data volume and the possible predominance of one data source over the others (Borras et al., 2015). In our case, models based on full HSI spectra had very satisfying classification results. However, after concatenating full HSI with full MIR or full LIBS, the accuracy of models has declined slightly when compared with results based on full HSI but increased obviously when compared with results based on full MIR or full LIBS. That indicated full HSI spectra had predominance over the other data sources. It should be addressed that the different sources of data can have a very different scale, and the appropriate preprocessing is of great importance before establishing models. Besides, there can exist some redundant information when using different instruments. In these conditions, it is critical to preprocess the raw data before data fusion, and sometimes fusion of fewer techniques might be able to obtain satisfactory results.

Mid-level fusion can partially overcome the high-data–volume problem. The data dimensionality could be significantly reduced with feature extraction methods. Besides, this fusion strategy is helpful to filter individual instrument noise and enable the interpretation of the results because of the fewer dimensionalities of inputs (Borras et al., 2015). Berdugo et al. (2014) adopted mid-level fusion with at least one feature per sensor among hyperspectral imaging, thermography, and chlorophyll fluorescence to detect cucumber disease. The most discriminant features from thermography and chlorophyll fluorescence had limited power to identify or differ plant diseases or abiotic stress. However, hyperspectral imaging was good at assessing disease-specific changes. Therefore, the features extracted from different instruments were cooperative, and the fusion of these features was helpful to filter noise existing in each instrument and obtain more complete information.

Furthermore, the fewer features within mid-level fusion were helpful to develop a real-time disease detection system. Moshou et al. (2005) assessed the real-time implementation of the Self-Organizing Map (SOM) neural network to detect wheat disease. The three selected spectral reflectance values were further fused with one fluorescence feature. The SOM classifier based on the fusion data achieved the overall classification accuracy of around 99%, which was higher than using one fluorescence feature. Besides, the fusion of features from different instruments may cause the issue of redundant information. To detect grape leaf disease, Adeel et al. (2019) implemented canonical correlation analysis for feature fusion and further performed neighborhood correlation analysis to reduce the dimensionalities and redundant information of the fused data before feeding the fused data into the classifier. This strategy helped to achieve an accuracy of 94.1% that was superior to the existing methods. In terms of disadvantages, mid-level fusion requires a preliminary feature extraction stage. Besides, taking account the many combinations of feature extraction methods and preprocessing, testing all the combinations makes the whole process cumbersome, computationally intensive, and difficult to validate (Borras et al., 2015).

Lastly, high-level fusion is operated with the classification results of individual classifiers. These separate models are developed based on the data of different instrumental techniques. Their predictions are then integrated into a single final response. Through a majority vote, a sample is assigned to the most-frequently predicted category. In this study involving four categories and three instrumental data, if the sample was predicted as class 1 by the two of the three classifiers, it would be assigned to class 1. Moreover, there are some other more complex protocols, such as Bayesian statistics (Biancolillo et al., 2014), which can be applied for decision making of high-level fusion. Concerning this type of fusion, every individual instrument is treated independently. Therefore, the responses from inefficient techniques (like LIBS spectra in this article) do not worsen the overall performance. Moreover, it is easy to add new techniques for final decision making when a new type of data is available. This increases the versatility of the decision-making process.

To better understand the differences across different methods, the ANOVA analysis was carried out. Diagnostic analysis among models was mainly discussed here, and the variance of rice variety and spectroscopic techniques was not considered. The influence of the type of input (full spectra, PCA features, and AE features) on classification results of CNN was analyzed. Besides, the difference of CNN based on different levels of fusion was also analyzed. On the one hand, the ANOVA analysis was performed on results based on full spectra (including Full-HSI, Full-MIR, and Full-LIBS), PCA features (including PCA-HSI, PCA-MIR, and PCA-LIBS), and AE features (including AE-HSI, AE-MIR, and AE-LIBS). The analysis results are summarized in Supplementary Table 1 (based on classification results of the training set), Supplementary Table 2 (based on classification results of the validation set), and Supplementary Table 3 (based on classification results of the test set), respectively. Since all CNN models achieved the accuracy of about 100% on the training set, all *p* values in Supplementary Table 1 were greater than 0.2, which indicated there was no obvious difference between classification results based on full spectra and those based on features. Similar results could also be found in Supplementary Table 2. However, Supplementary Table 3 showed that the significance value (*p* = 0.008) between classification results based on full spectra and classification results based on AE features was smaller than 0.05, which suggested classification results based on these two sources of data had significant differences. On the other hand, the ANOVA analysis was carried out on a different level of fusion, including nonfusion, low-level fusion, mid-level fusion, and high-level fusion. The ANOVA results are summarized in Supplementary Table 4 (based on classification results of the training set), Supplementary Table 5 (based on classification results of the validation set), and Supplementary Table 6 (based on classification results of the test set), respectively. The analysis results based on the training set and the validation set revealed most *p* values across two different groups were greater than 0.25, which suggested different levels of fusion had little effect on classification results. Besides, a *p* value equaled to 0.063 in Supplementary Table 6, which indicated the difference between classification results based on nonfusion and results based on mid-level fusion was not significant at the α = 0.05 level but was significant at the α = 0.1 level. In addition, another *p* value equaled to 0.055 in Supplementary Table 6, which indicated the difference between classification results based on mid-fusion and results based on high-level fusion was significant at the α = 0.1 level as well.

## Conclusion

In this study, HSI, MIR, and LIBS were applied to detect rice leaves inoculated by different diseases. Models based on full HSI spectra had the best performance among three full spectra. Based on full HSI, SVM, LR, and CNN obtained the accuracy of 90.38, 98.08, and 100% for the test set, respectively. PCA was an effective tool to extract key information. All three classifiers based on PCA-HSI obtained 94% accuracy for the test set. Besides, as part of the deep learning framework, AE was proved to be effective to extract features and reduce data dimension. Three kinds of data fusion strategies were explored for classification. The low-level fusion strategy was the least effective among the three fusion strategies due to the huge dimensions of fused data. Combined with appropriate feature extraction methods, the mid-level fusion exhibited better performance when compared with nonfused data. By integrating the PCA features of HSI and the PCA features of MIR, LR achieved an accuracy of over 98% for both rice varieties. Besides, it took less time to model with features. Overall, decision level fusion was a good way to avoid the limitation of decision making based on a single kind of classifier. In terms of the high-level fusion of classifiers based on full spectra, compared with the accuracy of SVM models based on full MIR, the accuracy of the test set after fusion was improved from 76.92 to 90.38% for Zhefujing83 and from 85.94 to 93.75% for AD516. Concerning high-level fusion of classifiers based on PCA features, the accuracy of both the training set and the validation set was increased to 100% compared with the accuracy based on PCA features of a single type of spectra. In terms of the high-level fusion of AE features, the accuracy of SVM was increased to 100% for both the training set and the validation set after data fusion, and the accuracy for the test set after fusion obtained 3.85 and 17.31% higher than that based on AE-HSI and AE-LIBS, respectively. In this work, CNN did not achieve excellent performance due to the limited number of samples in the training set (only 30 samples per category), but the great potential of CNN for rice diseases detection could be observed. More samples are required to make full use of CNN. More rapid and sensitive analytical techniques are available in industrial processes and laboratories, which will keep promoting advances in data fusion in various fields. There is still room for improvement in different levels of fusion.

## Data Availability Statement

The raw data supporting the conclusions of this article will be made available by the authors, without undue reservation.

## Author Contributions

LF, YH, and CZ: conceptualization. CZ and BW: data curation. JW and ZS: formal analysis. YH and LF: funding acquisition. CZ and JW: investigation. SZ, FL, ZS, and CZ: methodology. LF, CZ, and YH: project administration. CZ, JW, and ZS: resources. FL and CZ: software. CZ, LF, and SZ: supervision. SZ and CZ: validation. BW and LF: visualization. LF and BW: writing – original draft. CZ and YH: writing – review and editing. All authors contributed to the article and approved the submitted version.

## Conflict of Interest

The authors declare that the research was conducted in the absence of any commercial or financial relationships that could be construed as a potential conflict of interest.

## References

[B1] Adeel A. Khan M. A. Sharif M. Azam F. Shah J. H. Umer T. (2019). Diagnosis and recognition of grape leaf diseases: an automated system based on a novel saliency approach and canonical correlation analysis based multiple features fusion. *Sustain. Comput. Inform. Syst.* 24:100349 10.1016/j.suscom.2019.08.002

[B2] Alberto R. T. (2018). Spectral characterization of bacterial leaf blight of rice through spectroscopy and remotely sensed multi-spectral imagery. *Phytopathology* 108.

[B3] Altangerel N. Ariunbold G. O. Gorman C. Alkahtani M. H. Borrego E. J. Bohlmeyer D. (2017). In vivo diagnostics of early abiotic plant stress response via Raman spectroscopy. *Proc. Natl. Acad. Sci. U.S.A.* 114 3393–3396. 10.1073/pnas.1701328114 28289201 PMC5380084

[B4] Asaari M. S. M. Mishra P. Mertens S. Dhondt S. Inze D. Wuyts N. (2018). Close-range hyperspectral image analysis for the early detection of stress responses in individual plants in a high-throughput phenotyping platform. *ISPRS J. Photogram. Remote Sens.* 138 121–138. 10.1016/j.isprsjprs.2018.02.003

[B5] Ballabio D. Robotti E. Grisoni F. Quasso F. Bobba M. Vercelli S. (2018). Chemical profiling and multivariate data fusion methods for the identification of the botanical origin of honey. *Food Chem.* 266 79–89. 10.1016/j.foodchem.2018.05.084 30381229

[B6] Berdugo C. A. Zito R. Paulus S. Mahlein A. K. (2014). Fusion of sensor data for the detection and differentiation of plant diseases in cucumber. *Plant Pathol.* 63 1344–1356. 10.1111/ppa.12219

[B7] Biancolillo A. Bucci R. Magri A. L. Magri A. D. Marini F. (2014). Data-fusion for multiplatform characterization of an Italian craft beer aimed at its authentication. *Anal. Chim. Acta* 820 23–31. 10.1016/j.aca.2014.02.024 24745734

[B8] Borras E. Ferre J. Boque R. Mestres M. Acena L. Busto O. (2015). Data fusion methodologies for food and beverage authentication and quality assessment - a review. *Anal. Chim. Acta* 891 1–14. 10.1016/j.aca.2015.04.042 26388360

[B9] Carter G. A. Knapp A. K. (2001). Leaf optical properties in higher plants: linking spectral characteristics to stress and chlorophyll concentration. *Am. J. Bot.* 88 677–684. 10.2307/2657068 11302854

[B10] Castanedo F. (2013). A review of data fusion techniques. *Sci. World J.* 2013:704504. 10.1155/2013/704504 24288502 PMC3826336

[B11] Diniz P. Gomes A. A. Pistonesi M. F. Band B. S. F. de Araujo M. C. U. (2014). Simultaneous classification of teas according to their varieties and geographical origins by using NIR Spectroscopy and SPA-LDA. *Food Analyt. Methods* 7 1712–1718. 10.1007/s12161-014-9809-7

[B12] Doeswijk T. G. Smilde A. K. Hageman J. A. Westerhuis J. A. van Eeuwijk F. A. (2011). On the increase of predictive performance with high-level data fusion. *Anal. Chim. Acta* 705 41–47. 10.1016/j.aca.2011.03.025 21962346

[B13] Farber C. Mahnke M. Sanchez L. Kurouski D. (2019). Advanced spectroscopic techniques for plant disease diagnostics. A review. *Trac Trends Analyt. Chem.* 118 43–49. 10.1016/j.trac.2019.05.022

[B14] Feng L. Zhu S. Zhou L. Zhao Y. Bao Y. Zhang C. (2019). Detection of subtle bruises on winter jujube using hyperspectral imaging with pixel-wise deep learning method. *IEEE Access.* 7 64494–64505. 10.1109/access.2019.2917267

[B15] Feng L. Zhu S. S. Zhang C. Bao Y. D. Gao P. He Y. (2018). Variety identification of raisins using near-infrared hyperspectral imaging. *Molecules* 23:2907. 10.3390/molecules23112907 30412997 PMC6278444

[B16] Gaoqiang L. Changwen D. Fei M. Yazhen S. Jianmin Z. (2020). Responses of leaf cuticles to rice blast: detection and identification using depth-profiling fourier transform mid-infrared photoacoustic Spectroscopy. *Plant Dis.* 104 847–852. 10.1094/PDIS-05-19-1004-RE 31940445

[B17] Hawkins S. A. Park B. Poole G. H. Gottwald T. Windham W. R. Lawrence K. C. (2010). Detection of citrus huanglongbing by fourier transform infrared-attenuated total reflection Spectroscopy. *Appl. Spectrosc.* 64 100–103. 10.1366/000370210790572043 20132604

[B18] Knauer U. Matros A. Petrovic T. Zanker T. Scott E. S. Seiffert U. (2017). Improved classification accuracy of powdery mildew infection levels of wine grapes by spatial-spectral analysis of hyperspectral images. *Plant Methods* 13:47. 10.1186/s13007-017-0198-y 28630643 PMC5472862

[B19] Kumar A. Kumar R. Sengupta D. Das S. N. Pandey M. K. Bohra A. (2020). Deployment of genetic and genomic tools toward gaining a better understanding of rice-*Xanthomonasoryzae pv. oryzae* interactions for development of durable bacterial blight resistant rice. *Front. Plant Sci.* 11:1152. 10.3389/fpls.2020.01152 32849710 PMC7417518

[B20] Lin Z. H. Chen Y. H. Zhao X. Wang G. (2013). “Spectral-spatial classification of hyperspectral image using autoencoders”, *Proceedings of the 2013 9th International Conference on Information, Communications & Signal Processing*, Tainan 10.1109/ICICS.2013.6782778

[B21] Liu F. Shen T. Wang J. He Y. Zhang C. Zhou W. (2019). Detection of sclerotinia stem rot on oilseed rape (*Brassica napus* L.) based on laser- induced breakdown spectroscopy. *Trans. Asabe* 62 123–130. 10.13031/trans.12206

[B22] Luo Y. H. Jiang P. Xie K. Wang F. J. (2019). Research on optimal predicting model for the grading detection of rice blast. *Opt. Rev.* 26 118–123. 10.1007/s10043-018-0487-3

[B23] Machado J. C. Faria M. A. Ferreira I. M. P. L. V. O. Páscoa R. N. M. J. Lopes J. A. (2018). Varietal discrimination of hop pellets by near and mid infrared spectroscopy. *Talanta* 180 69–75. 10.1016/j.talanta.2017.12.030 29332835

[B24] Molla K. A. Karmakar S. Molla J. Bajaj P. Varshney R. K. Datta S. K. (2020). Understanding sheath blight resistance in rice: the road behind and the road ahead. *Plant Biotechnol. J.* 18 895–915. 10.1111/pbi.13312 31811745 PMC7061877

[B25] Moshou D. Bravo C. Oberti R. West J. Bodria L. McCartney A. (2005). Plant disease detection based on data fusion of hyper-spectral and multi-spectral fluorescence imaging using Kohonen maps. *Real Time Imag.* 11 75–83. 10.1016/j.rti.2005.03.003

[B26] Ng W. Minasny B. McBratney A. (2020). Convolutional neural network for soil microplastic contamination screening using infrared spectroscopy. *Sci. Total Environ.* 702:134723. 10.1016/j.scitotenv.2019.134723 31731131

[B27] Peng J. Liu F. Zhou F. Song K. Zhang C. Ye L. (2016). Challenging applications for multi-element analysis by laser-induced breakdown spectroscopy in agriculture: a review. *TrAC Trends Analyt. Chem.* 85 260–272. 10.1016/j.trac.2016.08.015

[B28] Ponce L. Etxeberria E. Gonzalez P. Ponce A. Flores T. (2018). Rapid identification of Huan ongbing-infected citrus plants using laser-induced breakdown spectroscopy of phloem samples. *Appl. Opt.* 57 8841–8844. 10.1364/ao.57.008841 30461866

[B29] Ribeiro L. D. Klock A. L. S. Wordell J. A. Tramontin M. A. Trapp M. A. Mithofer A. (2018). Hyperspectral imaging to characterize plant-plant communication in response to insect herbivory. *Plant Methods* 14:54. 10.1186/s13007-018-0322-7 29988987 PMC6034322

[B30] Skolik P. McAinsh M. R. Martin F. L. (2019). ATR-FTIR spectroscopy non-destructively detects damage-induced sour rot infection in whole tomato fruit. *Planta* 249 925–939. 10.1007/s00425-018-3060-1 30488286

[B31] Sun J. Tang K. Wu X. H. Dai C. X. Chen Y. Shen J. F. (2018). Nondestructive identification of green tea varieties based on hyperspectral imaging technology. *J. Food Process Eng.* 41:e12800 10.1111/jfpe.12800

[B32] Thomas S. Behmann J. Steier A. Kraska T. Muller O. Rascher U. (2018). Quantitative assessment of disease severity and rating of barley cultivars based on hyperspectral imaging in a non-invasive, automated phenotyping platform. *Plant Methods* 14:45. 10.1186/s13007-018-0313-8 29930695 PMC5994119

[B33] Tremblay M. Kammer M. Lange H. Plattner S. Baumgartner C. Stegeman J. A. (2019). Prediction model optimization using full model selection with regression trees demonstrated with FTIR data from bovine milk. *Prevent. Vet. Med.* 163 14–23. 10.1016/j.prevetmed.2018.12.012 30670181

[B34] Wang L. Sun D.-W. Pu H. Zhu Z. (2016). Application of hyperspectral imaging to discriminate the variety of maize seeds. *Food Analyt. Methods* 9 225–234. 10.1007/s12161-015-0160-4

[B35] Wu N. Zhang C. Bai X. L. Du X. Y. He Y. (2018). Discrimination of chrysanthemum varieties using hyperspectral imaging combined with a deep convolutional neural network. *Molecules* 23:2831. 10.3390/molecules23112831 30384477 PMC6278476

[B36] Xing C. Ma L. Yang X. (2016). Stacked denoise autoencoder based feature extraction and classification for hyperspectral images. *J. Sensors* 2016:3632943 10.1155/2016/3632943

[B37] Yang J. Chen G. Li L. Zou L. Zhang C. Mao U. (2019). Portable rice disease spores capture and detection method using diffraction fingerprints on microfluidic chip. *Micromachines* 10:289. 10.3390/mi10050289 31035416 PMC6562855

[B38] Yuan C. Yuxiang Z. Zhijuan J. Yan L. Zhihua W. Changdeng Y. (2019). Identification of stable quantitative trait loci for sheath blight resistance using recombinant inbred line. *Rice Sci.* 26 331–338. 10.1016/j.rsci.2019.08.007

[B39] Zabalza J. Ren J. Zheng J. Zhao H. Qing C. Yang Z. (2016). Novel segmented stacked autoencoder for effective dimensionality reduction and feature extraction in hyperspectral imaging. *Neurocomputing* 185 1–10. 10.1016/j.neucom.2015.11.044

[B40] Zahid A. Abbas H. T. Ren A. Zoha A. Heidari H. Shah S. A. (2019). Machine learning driven non-invasive approach of water content estimation in living plant leaves using terahertz waves. *Plant Methods* 15:138. 10.1186/s13007-019-0522-9 31832080 PMC6859614

[B41] Zhang C. Feng X. Wang J. Liu F. He Y. Zhou W. (2017). Mid-infrared spectroscopy combined with chemometrics to detect *Sclerotinia* stem rot on oilseed rape (*Brassica napus* L.) leaves. *Plant Methods* 13:39. 10.1186/s13007-017-0190-6 28529536 PMC5436460

[B42] Zhang C. Liu F. He Y. (2018). Identification of coffee bean varieties using hyperspectral imaging: influence of preprocessing methods and pixel-wise spectra analysis. *Sci. Rep.* 8:11. 10.1038/s41598-018-20270-y 29391427 PMC5794930

[B43] Zhang C. Wu W. Y. Zhou L. Cheng H. Ye X. Q. He Y. (2020). Developing deep learning based regression approaches for determination of chemical compositions in dry black goji berries (*Lycium ruthenicum* Murr.) using near-infrared hyperspectral imaging. *Food Chem.* 319:126536. 10.1016/j.foodchem.2020.126536 32146292

[B44] Zhou L. Zhang C. Qiu Z. He Y. (2020). Information fusion of emerging non-destructive analytical techniques for food quality authentication: a survey. *TrAC Trends Analyt. Chem.* 127:115901 10.1016/j.trac.2020.115901

[B45] Zhu S. Feng L. Zhang C. Bao Y. He Y. (2019). Identifying freshness of spinach leaves stored at different temperatures using hyperspectral imaging. *Foods* 8:356. 10.3390/foods8090356 31438644 PMC6770342

[B46] Zhu S. S. Zhou L. Gao P. Bao Y. D. He Y. Feng L. (2019). Near-infrared hyperspectral imaging combined with deep learning to identify cotton seed varieties. *Molecules* 24:3268. 10.3390/molecules24183268 31500333 PMC6766998

