# Supplementary material for: Investigation on Data Fusion of Multisource Spectral Data for Rice Leaf Diseases Identification Using Machine Learning Methods
Source: Front Plant Sci. 2020 Nov 10;11:577063. doi: 10.3389/fpls.2020.577063 (PMC7683421; doi:10.3389/fpls.2020.577063)
Supplement: Supplementary file 1 [file Data_Sheet_1.docx]

Supplementary Material

# Supplementary Figures and Tables

## Supplementary Figures

| 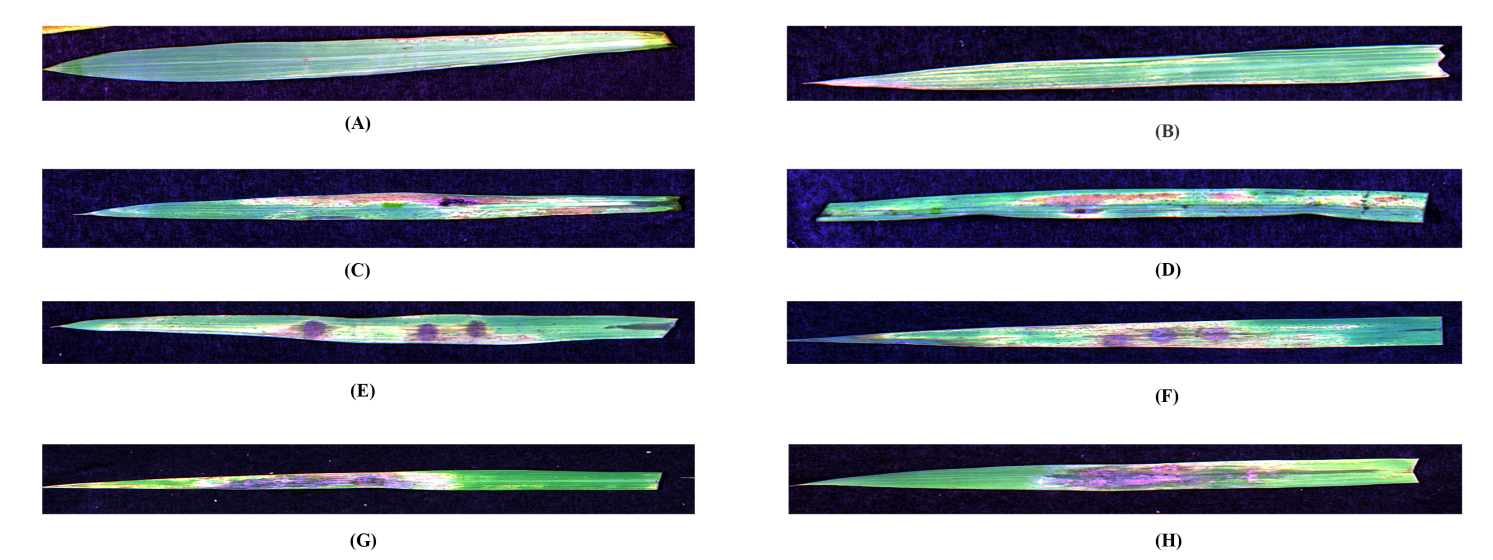 |
| --- |

**Supplementary Figure 1.** **(A)** CK of Zhefujing83; **(B)** CK of AD516; **(C)** BYK of Zhefujing83; **(D)** BYK of AD516; **(E)** DWB of Zhefujing83; **(F)** DWB of AD516; (G) WKB of Zhefujing83; **(H)** WKB of AD516.

| **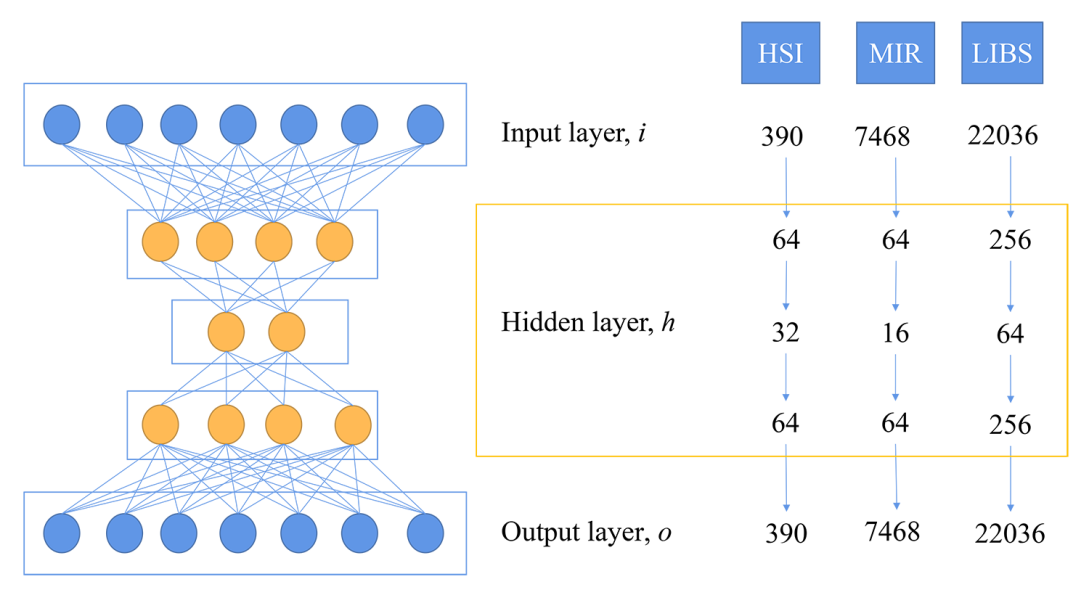** |
| --- |

**Supplementary Figure 2.** The architecture of autoencoder. For HSI, the data dimensionalities from beginning to the end could be simply recorded as 390-64-32-64-390. For MIR, the change of dimensionalities could be simply recorded as 7468-64-16-64-7468. For LIBS, the change of dimensionalities could be simply recorded as 22036-256-64-256-22036.

| 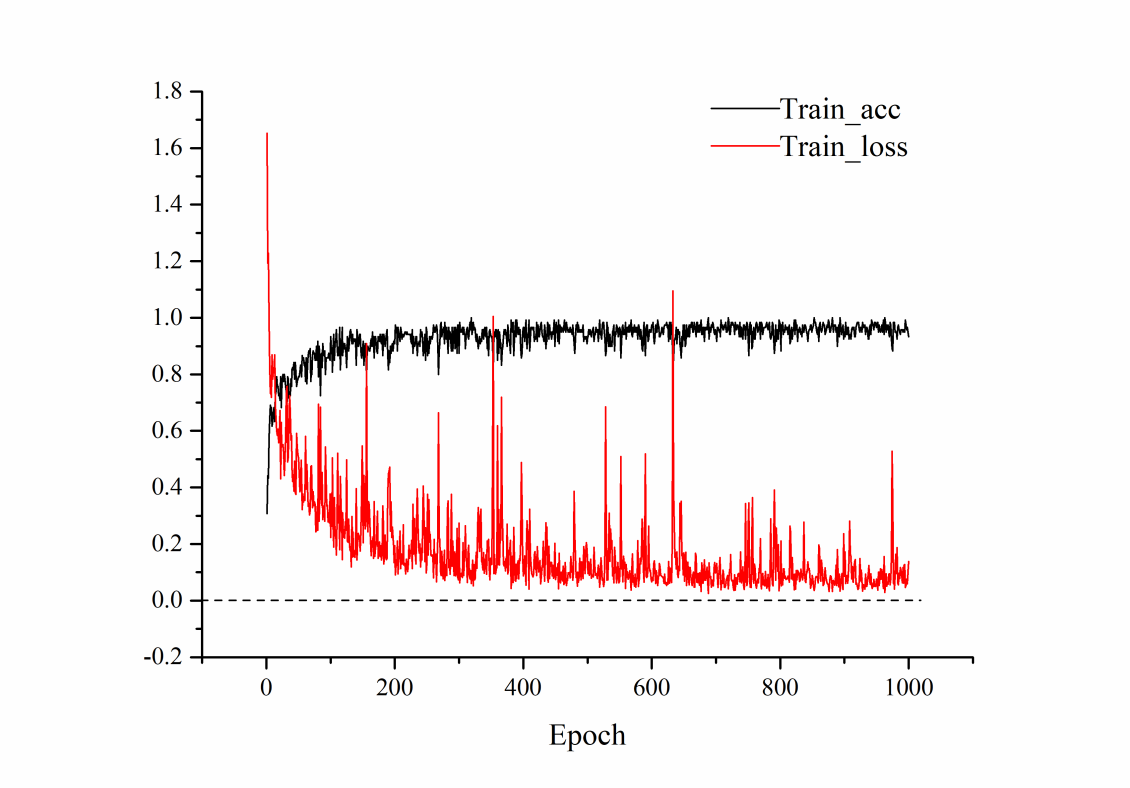 |
| --- |

**Supplementary Figure 3.** The relationship between epoch and training performances.

| 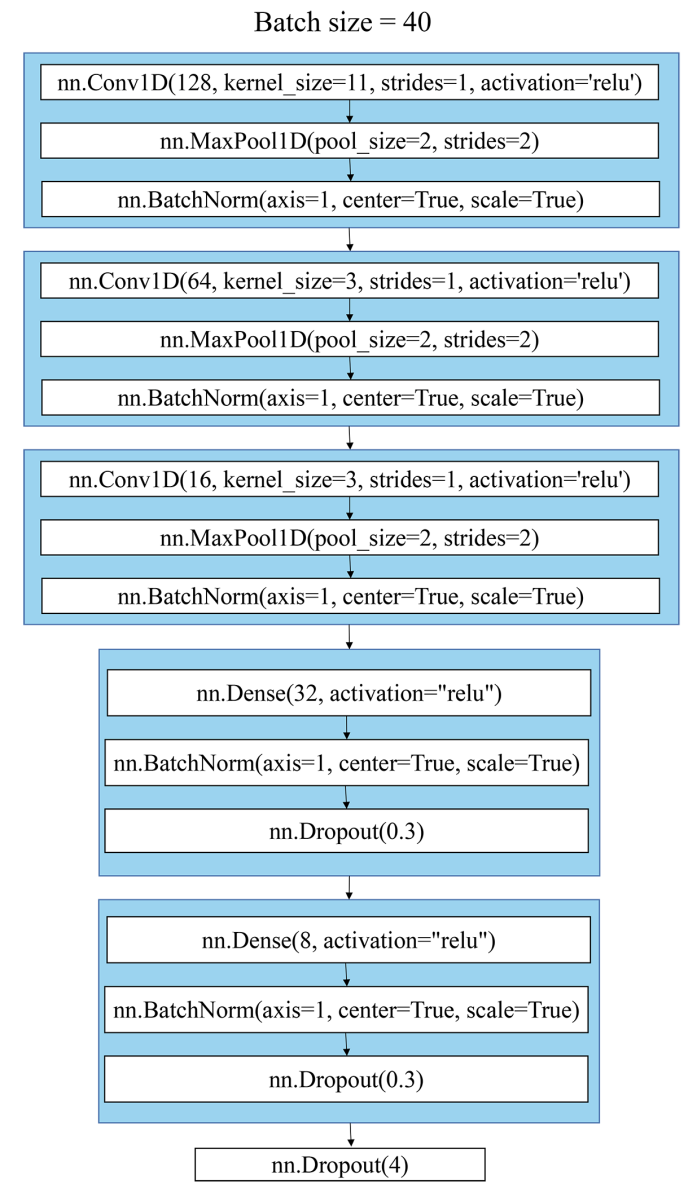 |
| --- |

**Supplementary Figure 4.** The CNN architecture of Full-HSI of Zhefujing83 and AD516.

| 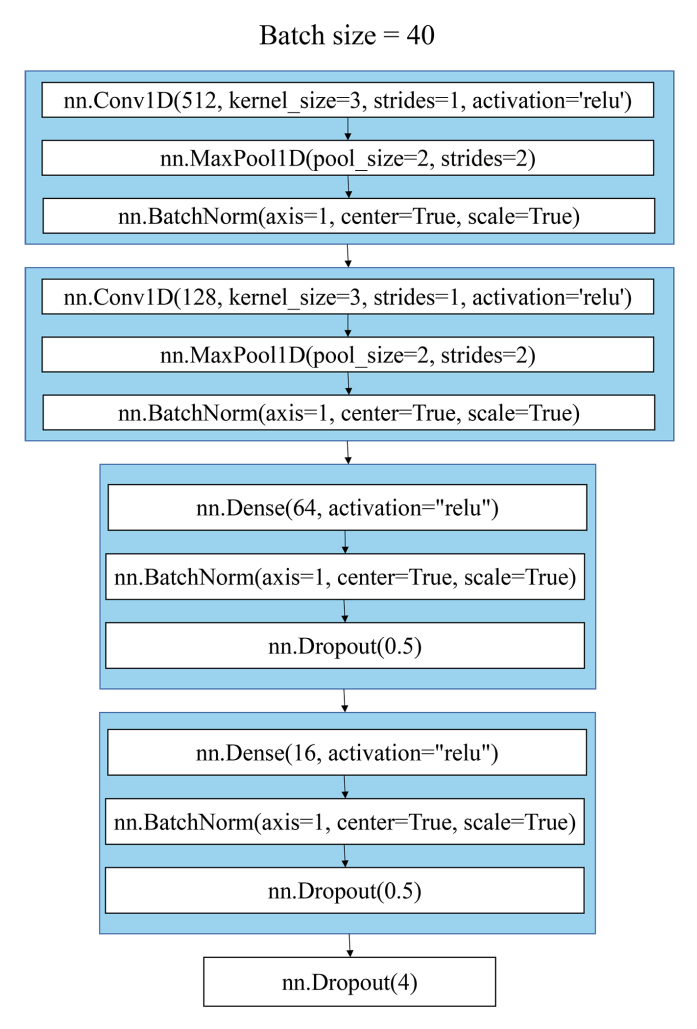 |
| --- |

**Supplementary Figure 5.** The CNN architecture of Full-MIR of Zhefujing83 and AD516.

| 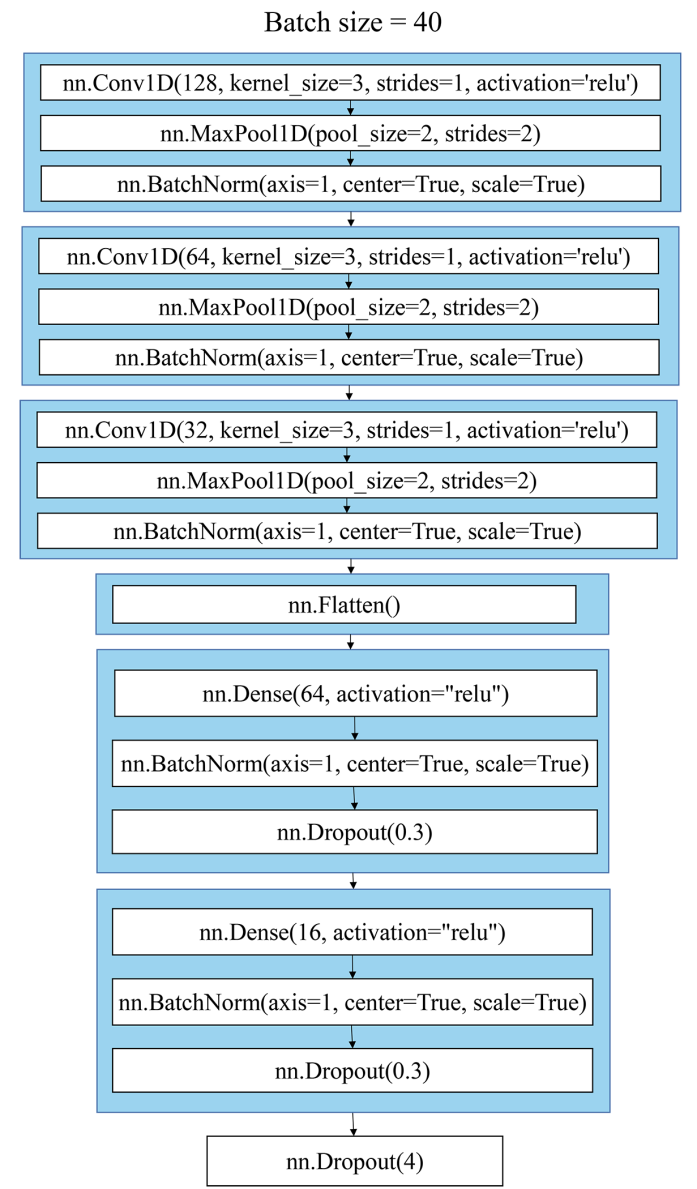 |
| --- |

**Supplementary Figure 6.** The CNN architecture of Full-LIBS of Zhefujing83 and AD516.

| 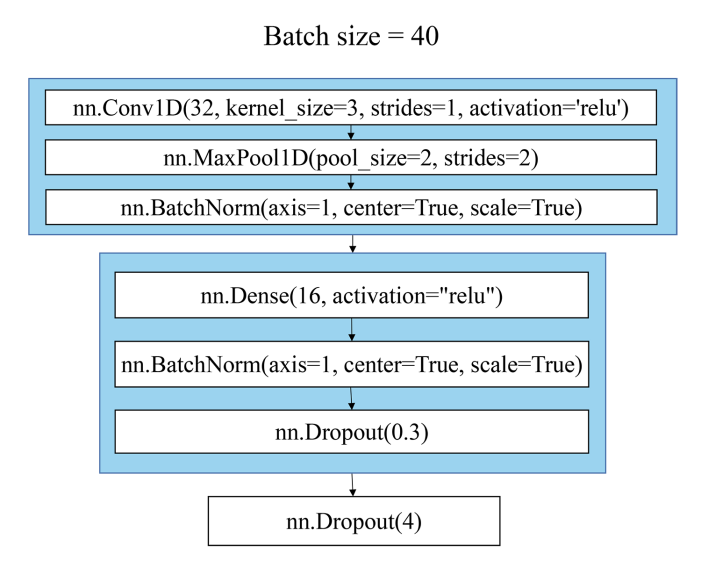**(A)** | 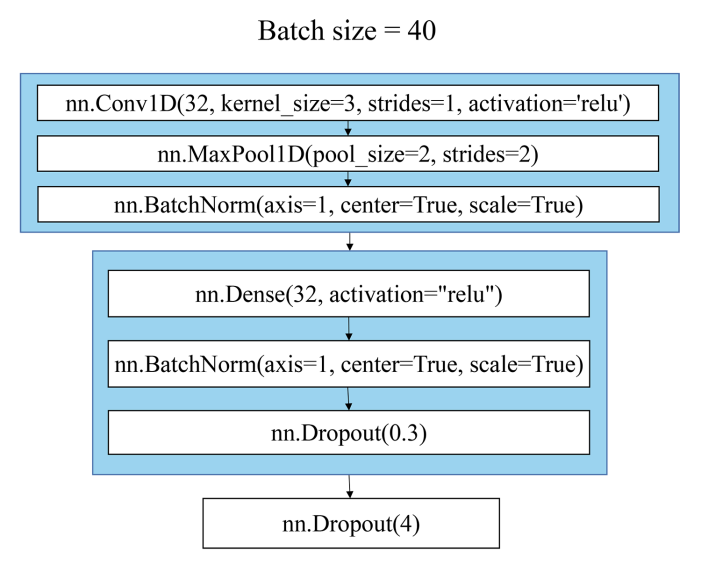**(B)** |
| --- | --- |

**Supplementary Figure 7.** **(A)** The CNN architecture of PCA-HSI of Zhefujing83; **(B)** The CNN architecture of PCA-HSI of AD516.

| 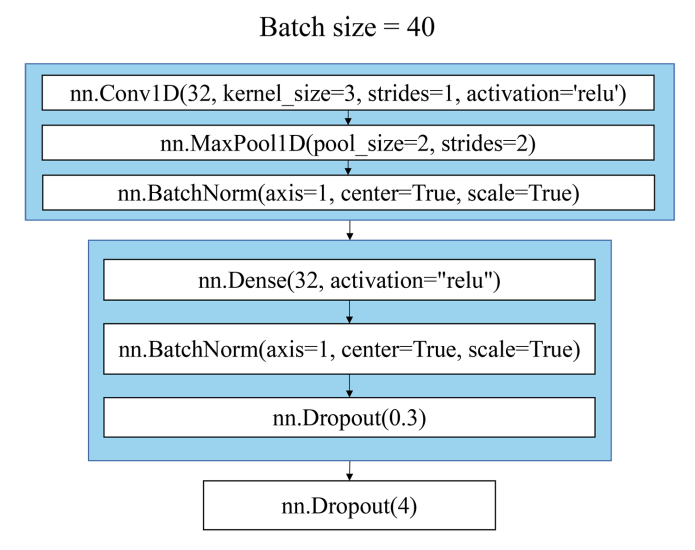(A) | 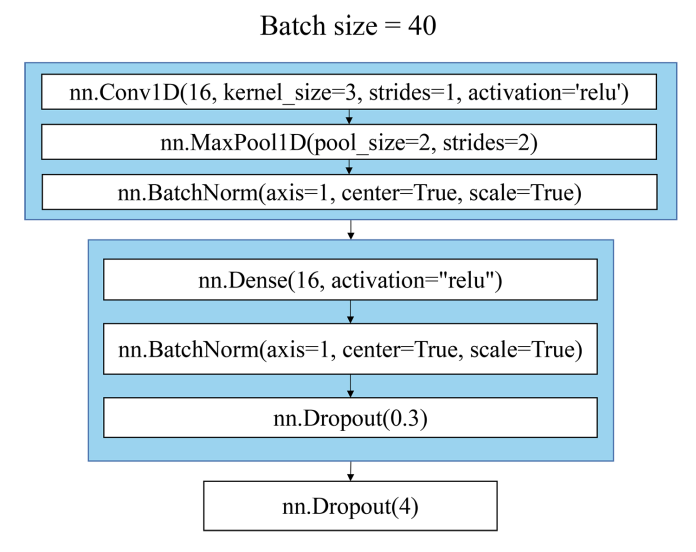**(B)** |
| --- | --- |

**Supplementary Figure 8.** **(A)** The CNN architecture of PCA-MIR of Zhefujing83; (**B)** The CNN architecture of PCA-MIR of AD516.

| 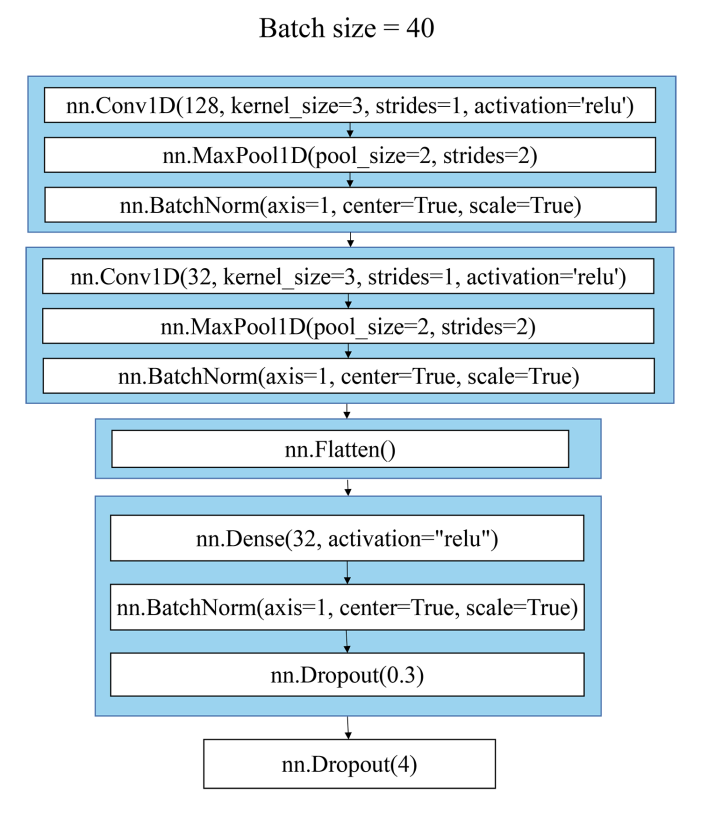**(A)** | 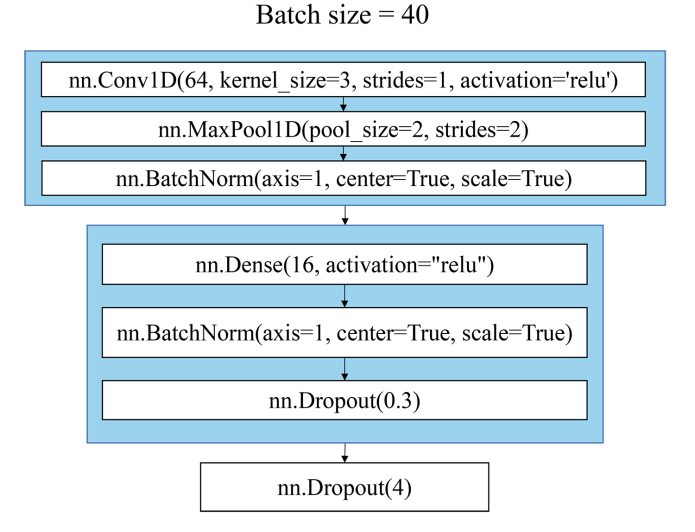  **(B)** |
| --- | --- |

**Supplementary Figure 9.** **(A)** The CNN architecture of PCA-LIBS of Zhefujing83; **(B)** the CNN architecture of PCA-LIBS of AD516.

| 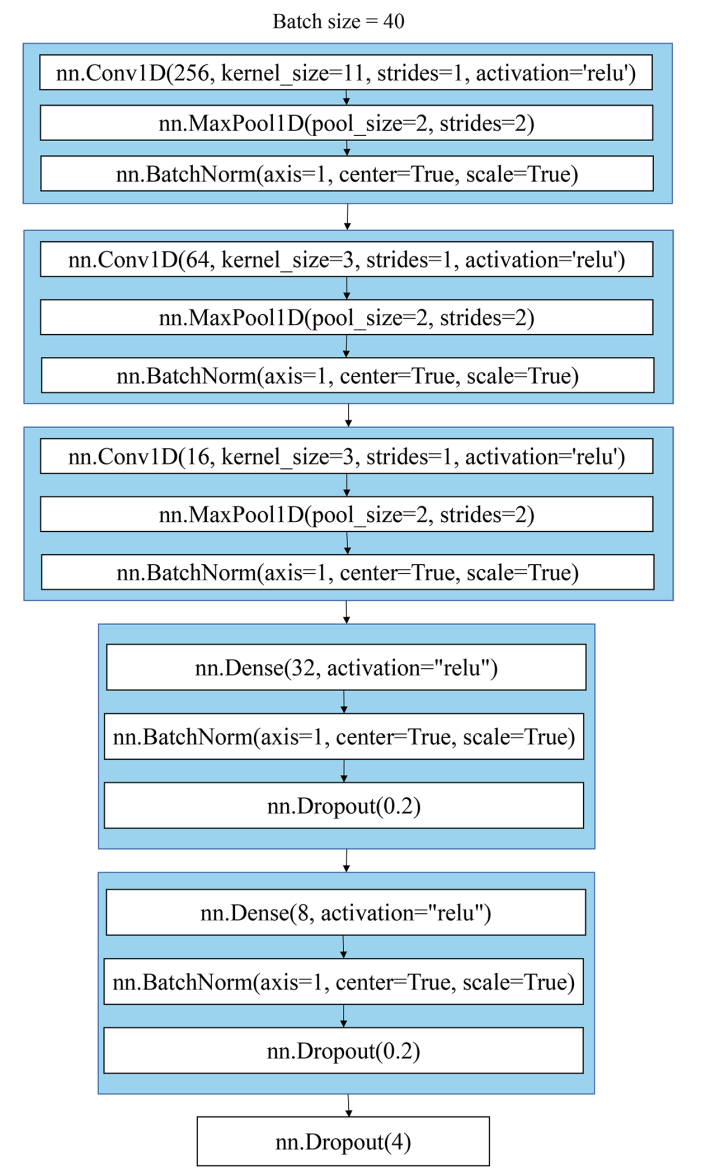 |
| --- |

**Supplementary Figure 10.** The CNN architecture of AE-HSI of Zhefujing83 and AD516.

| 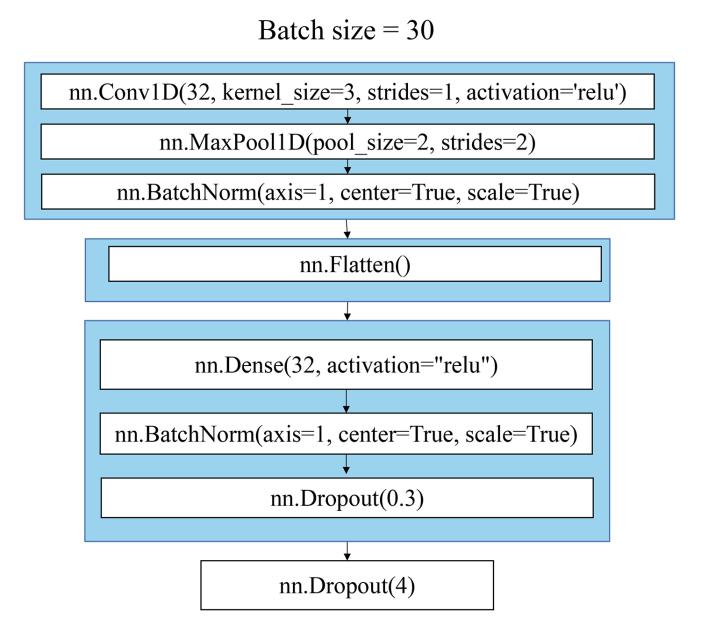 |
| --- |

**Supplementary Figure 11.** The CNN architecture of AE-MIR of Zhefujing83 and AD516.

| 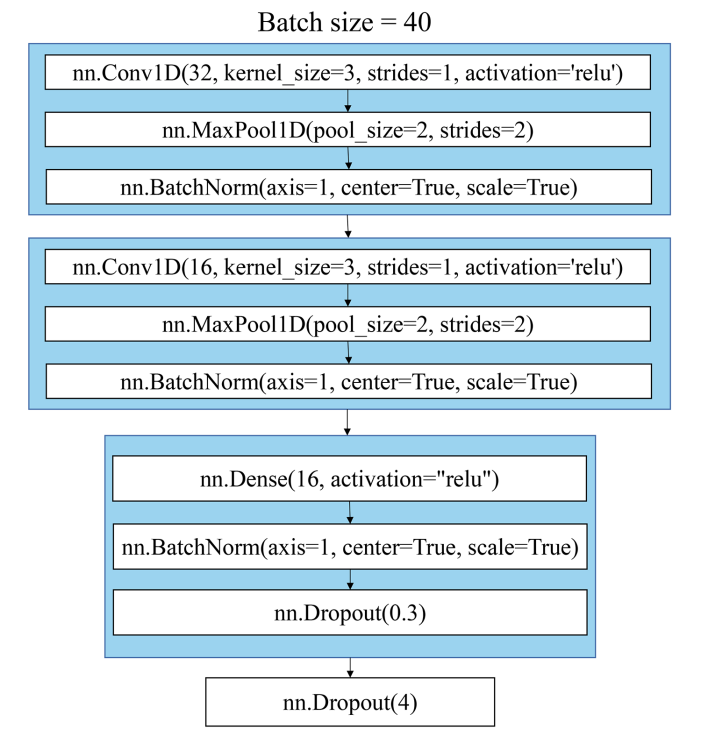  **(A)** | 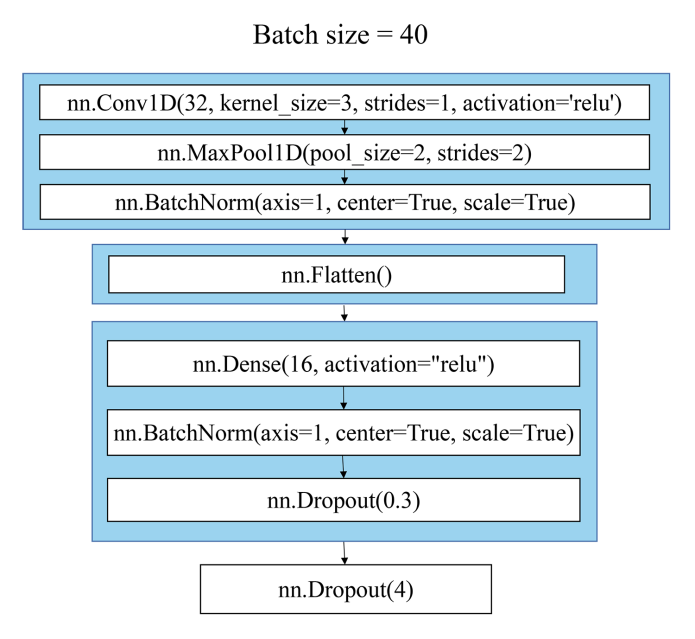  **(B)** |
| --- | --- |

**Supplementary Figure 12.** **(A)** The CNN architecture of AE-LIBS of Zhefujing83; **(B)** The CNN architecture of AE-LIBS of AD516.

| 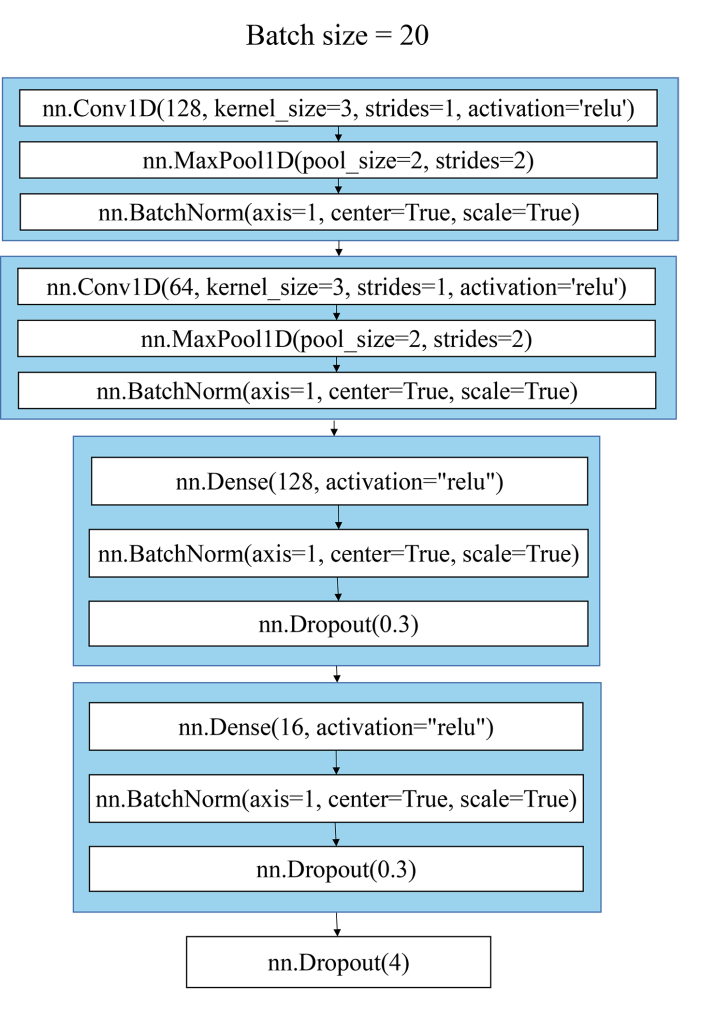 |
| --- |

**Supplementary Figure 13.** The CNN architecture of Full-HSI-MIR of Zhefujing83 and AD516.

| 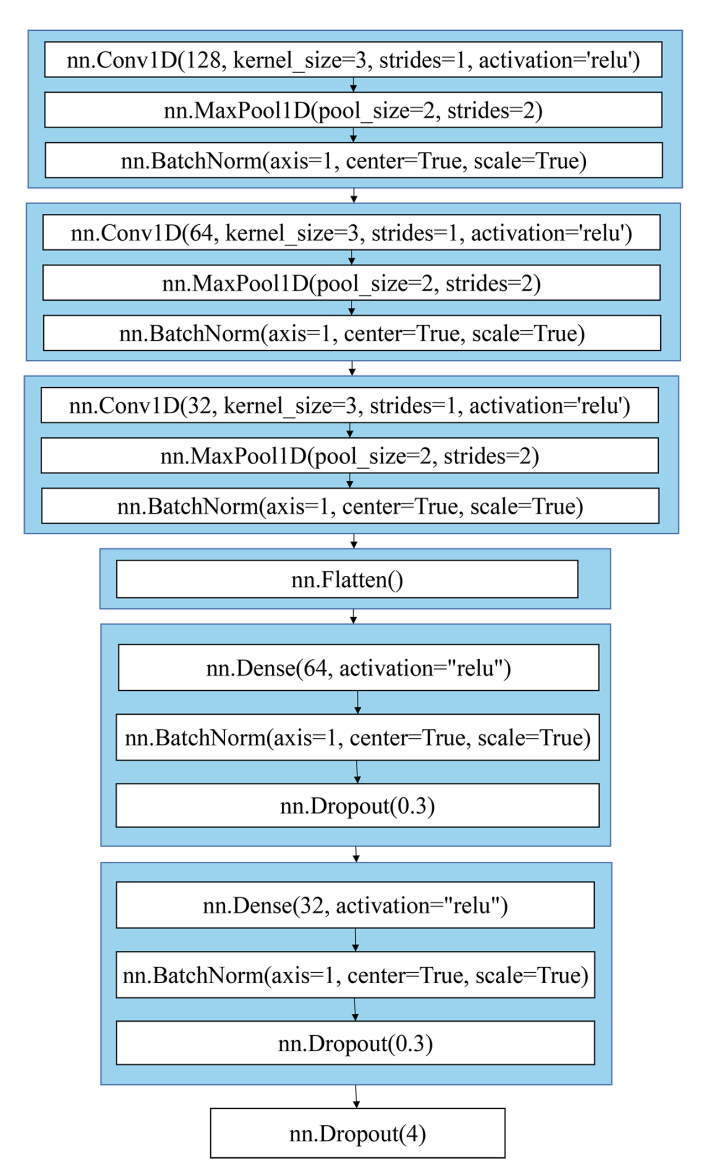 |
| --- |

**Supplementary Figure 14.** The CNN architecture of Full-HSI-LIBS (Batch size=20), Full-MIR-LIBS (Batch size=20) and Full-HSI-MIR-LIBS (Batch size=10) of Zhefujing83 and AD516.

| 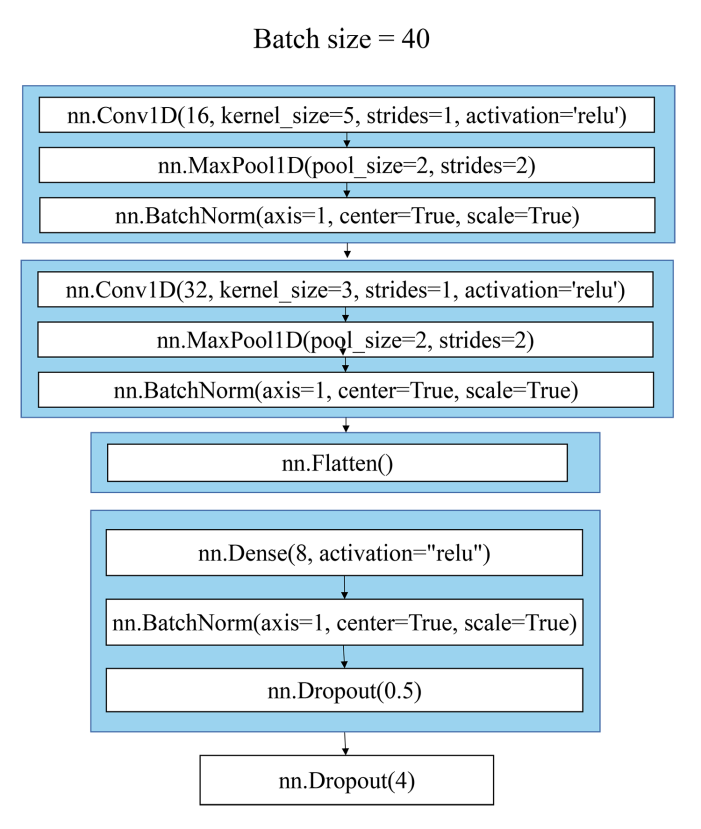 |
| --- |

**Supplementary Figure 15.** The CNN architecture of AE-PCA-HSI-MIR, AE-PCA-HSI-LIBS, AE-PCA-MIR-LIBS and AE-PCA-HSI-MIR-LIBS of Zhefujing83 and AD516.

| 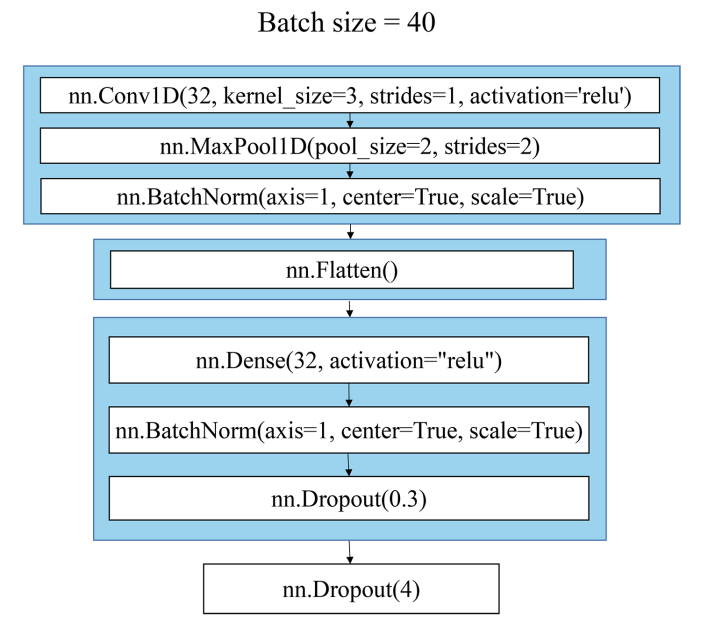  **(A)** | 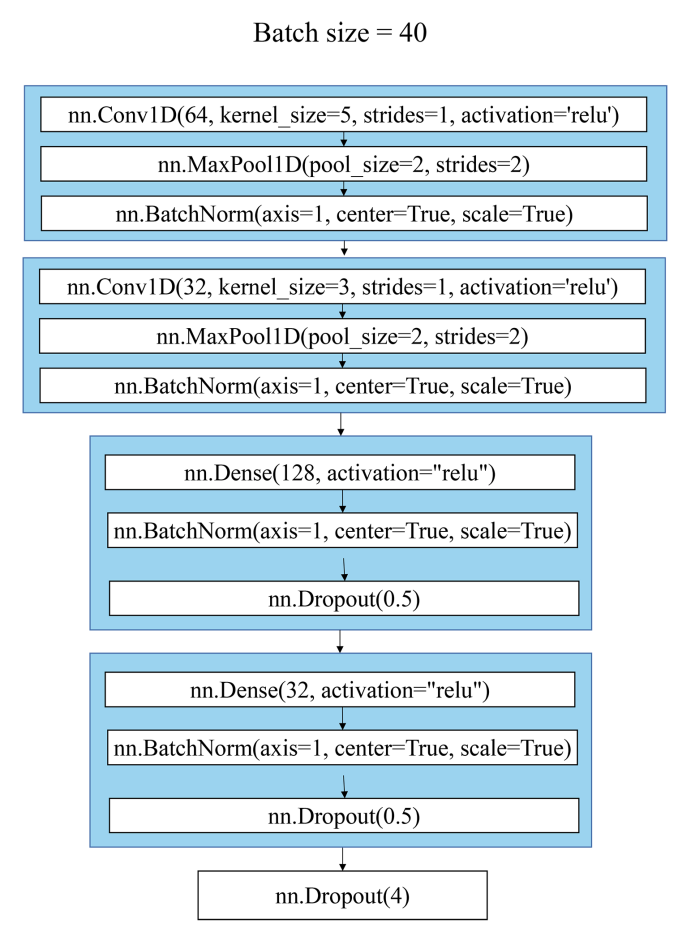  **(B)** |
| --- | --- |

**Supplementary Figure 16.** **(A)** The CNN architecture of PCA-HSI-MIR of Zhefujing83; **(B)** The CNN architecture of PCA-HSI-MIR of AD516.

| 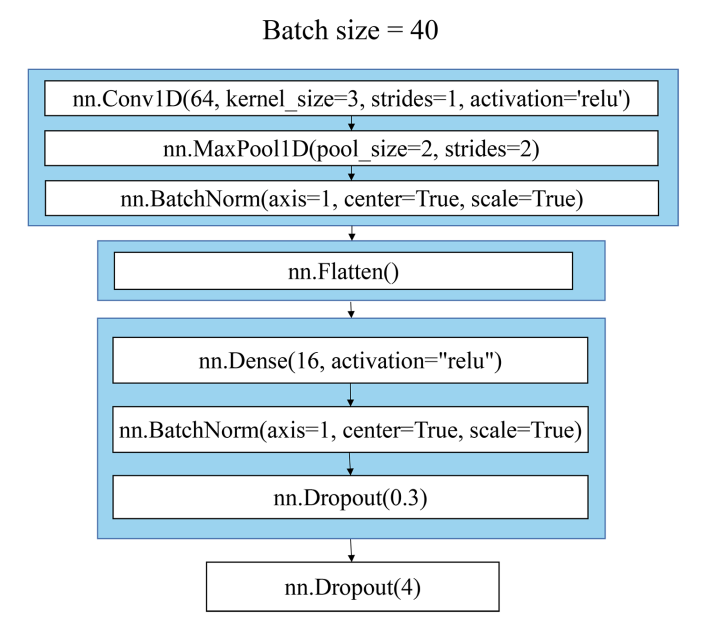  **(A)** | 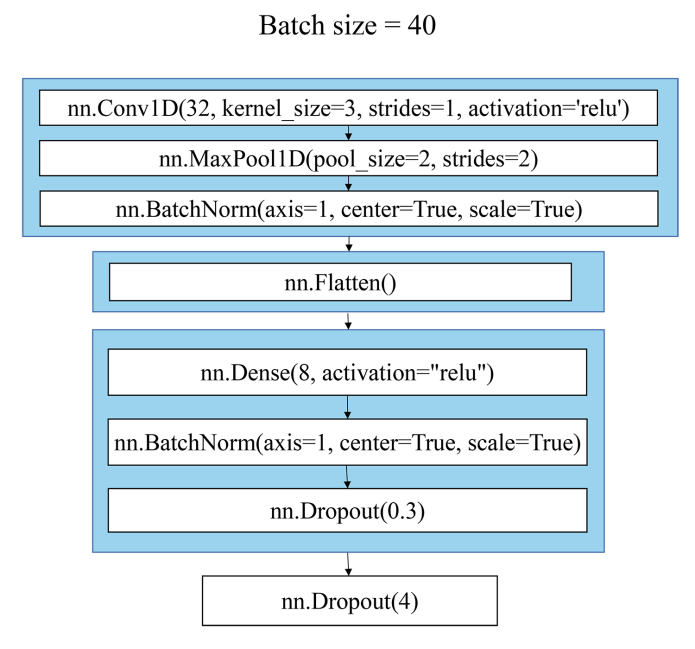  **(B)** |
| --- | --- |

**Supplementary Figure 17. (A)** The CNN architecture of PCA-HSI-LIBS of Zhefujing83; **(B)** The CNN architecture of PCA-HSI-LIBS of AD516.

| 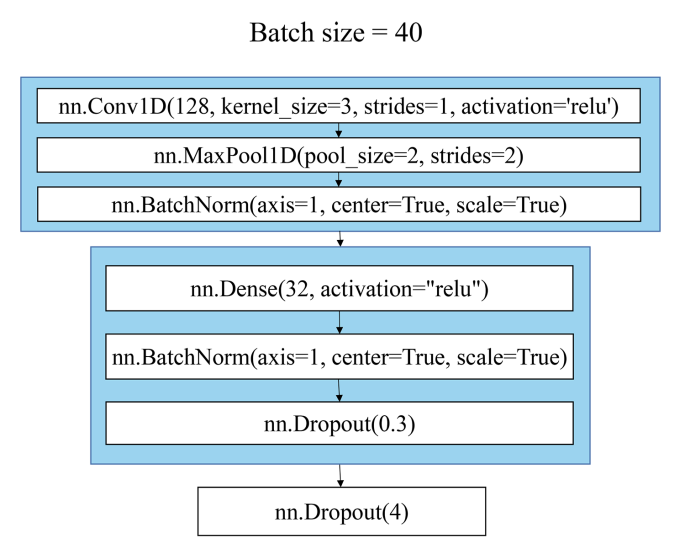  (A) | 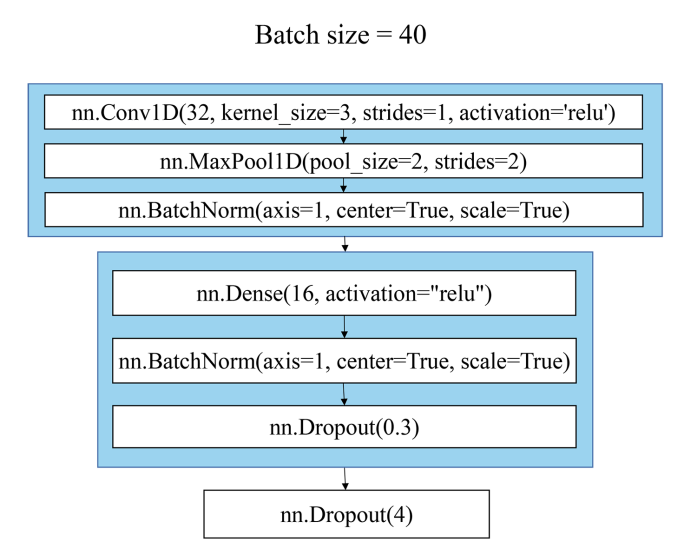  (B) |
| --- | --- |

**Supplementary Figure 18.** **(A)** The CNN architecture of PCA-MIR-LIBS of Zhefujing83; **(B)** The CNN architecture of PCA-MIR-LIBS of AD516.

| 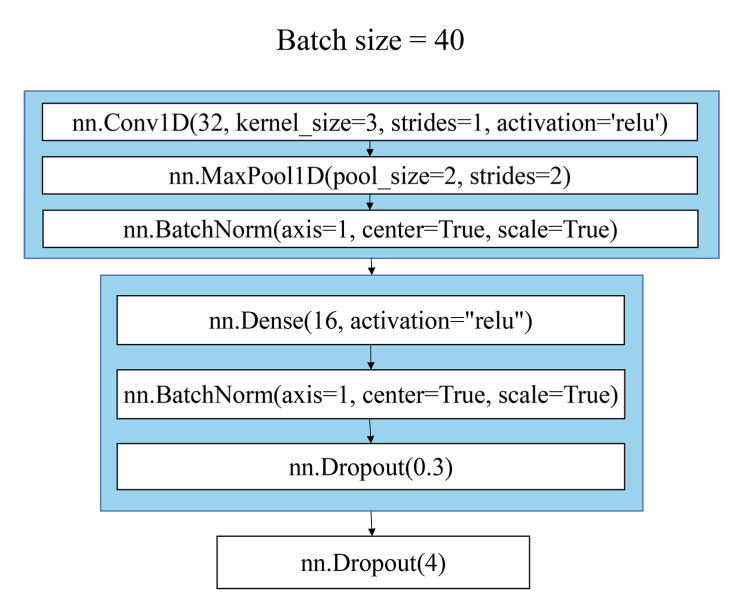 |
| --- |

**Supplementary Figure 19.** The CNN architecture of PCA-HSI-MIR-LIBS of Zhefujing83 and AD516.

| 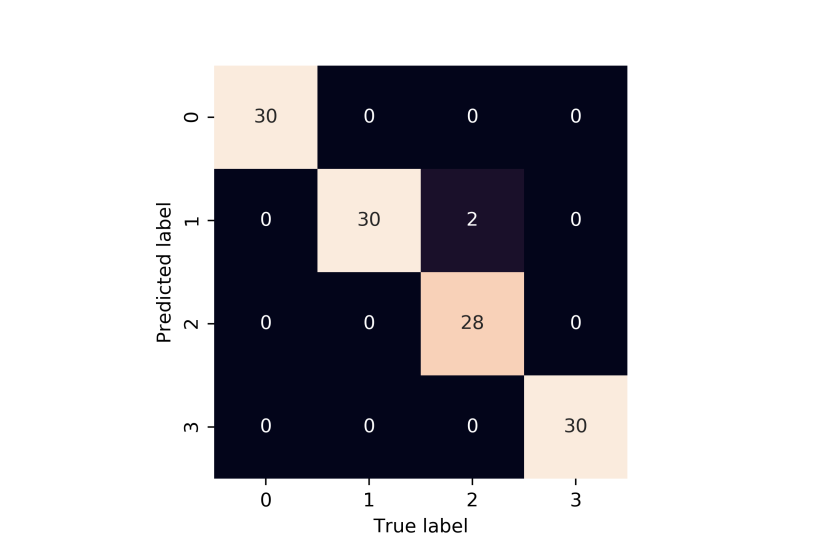  (A) | 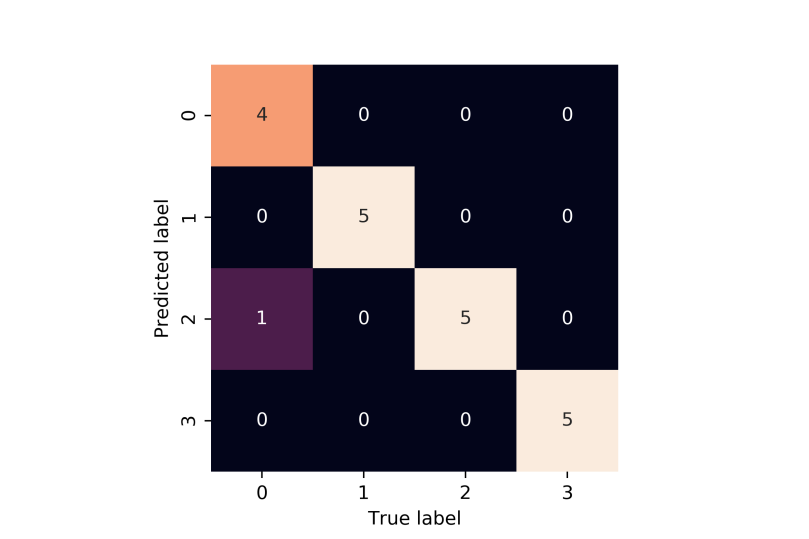  **(B)** |
| --- | --- |
| 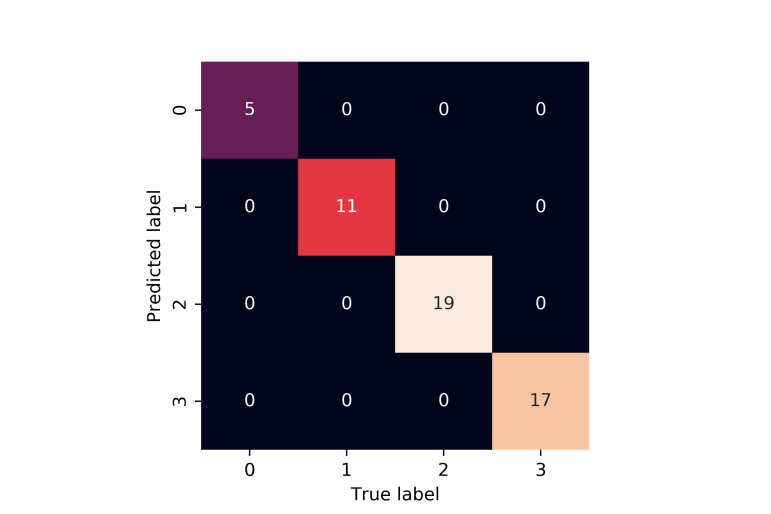  **(C)** | |

**Supplementary Figure 20.** The confusion matrix of the training set **(A)**, the validation set **(B)** and the test set **(C)** of Full-HSI of Zhefujing83.

**Supplementary Table 1.** ANOVA analysis of results of the training set of CNN for Full spectra, PCA features and AE features.

| Sig. | 0^a^ | 1^b^ | 2^c^ |
| --- | --- | --- | --- |
| 0 | - | 0.648 | 0.294 |
| 1 | 0.648 | - | 0.544 |
| 2 | 0.294 | 0.544 | - |

^a^ accuracy of training set of Full spectra; ^b^ accuracy of training set of PCA features; ^c^ accuracy of training set of AE features; Sig. means significance.

**Supplementary Table 2.** ANOVA analysis of results of the validation set of CNN Full spectra, PCA features and AE features.

| Sig. | 0^a^ | 1^b^ | 2^c^ |
| --- | --- | --- | --- |
| 0 | - | 0.801 | 0.093 |
| 1 | 0.801 | - | 0.145 |
| 2 | 0.093 | 0.145 | - |

^a^ accuracy of validation set of Full spectra; ^b^ accuracy of validation set of PCA features; ^c^ accuracy of validation set of AE features; Sig. means significance.

**Supplementary Table 3.** ANOVA analysis of results of the test set of CNN for Full spectra, PCA features and AE features.

| Sig. | 0^a^ | 1^b^ | 2^c^ |
| --- | --- | --- | --- |
| 0 | - | 0.709 | 0.008 |
| 1 | 0.709 | - | 0.017 |
| 2 | 0.008 | 0.017 | - |

^a^ accuracy of test set of Full spectra; ^b^ accuracy of test set of PCA features; ^c^ accuracy of test set of AE features; Sig. means significance.

**Supplementary Table 4.** ANOVA analysis of results of the training set of CNN for Full individual spectra, low-level fusion, mid-level fusion and high-level fusion.

| Sig. | 0^a^ | 1^b^ | 2^c^ | 3^d^ |
| --- | --- | --- | --- | --- |
| 0 | - | 0.163 | 0.232 | 0.191 |
| 1 | 0.163 | - | 0.615 | 1.0 |
| 2 | 0.232 | 0.615 | - | 0.652 |
| 3 | 0.191 | 1.0 | 0.652 | - |

^a^ accuracy of training set of Full individual spectra; ^b^ accuracy of training set of low-level fusion; ^c^ accuracy of training set of mid-level fusion; ^d^ accuracy of training set of high-level fusion; Sig. means significance.

**Supplementary Table 5.** ANOVA analysis of results of the validation set of CNN for Full individual spectra, low-level fusion, mid-level fusion and high-level fusion.

| Sig. | 0^a^ | 1^b^ | 2^c^ | 3^d^ |
| --- | --- | --- | --- | --- |
| 0 | - | 0.338 | 0.512 | 0.472 |
| 1 | 0.338 | - | 0.052 | 0.847 |
| 2 | 0.512 | 0.052 | - | 0.124 |
| 3 | 0.472 | 0.847 | 0.124 | - |

^a^ accuracy of validation set of Full individual spectra; ^b^ accuracy of validation set of low-level fusion; ^c^ accuracy of validation set of mid-level fusion; ^d^ accuracy of validation set of high-level fusion; Sig. means significance.

**Supplementary Table 6.** ANOVA analysis of results of the test set of CNN for Full individual spectra, low-level fusion, mid-level fusion and high-level fusion.

| Sig. | 0^a^ | 1^b^ | 2^c^ | 3^d^ |
| --- | --- | --- | --- | --- |
| 0 | - | 0.628 | 0.063 | 0.960 |
| 1 | 0.628 | - | 0.141 | 0.591 |
| 2 | 0.063 | 0.141 | - | 0.055 |
| 3 | 0.960 | 0.591 | 0.055 | - |

^a^ accuracy of test set of Full individual spectra; ^b^ accuracy of test set of low-level fusion; ^c^ accuracy of test set of mid-level fusion; ^d^ accuracy of test set of high-level fusion; Sig. means significance.
